# Exploring the impact of data curation criteria on the observed geographical distribution of mosses

**DOI:** 10.1002/ece3.10786

**Published:** 2023-12-03

**Authors:** Cristina Ronquillo, Juliana Stropp, Nagore G. Medina, Joaquin Hortal

**Affiliations:** ^1^ Department of Biogeography and Global Change Museo Nacional de Ciencias Naturales (MNCN‐CSIC) Madrid Spain; ^2^ Escuela Internacional de Doctorado Universidad Rey Juan Carlos (URJC) Madrid Spain; ^3^ Department of Biogeography Trier University Trier Germany; ^4^ Department of Biología (Botánica), Facultad de Ciencias Universidad Autónoma de Madrid Madrid Spain; ^5^ Centro de Investigación en Biodiversidad y Cambio Global (CIBC‐UAM), Facultad de Ciencias Universidad Autónoma de Madrid Madrid Spain

**Keywords:** biodiversity records, bryopsida, data curation, latitudinal gradient, mosses, species distributions

## Abstract

Biodiversity data records contain inaccuracies and biases. To overcome this limitation and establish robust geographic patterns, ecologists often curate records keeping those that are most suitable for their analyses. Yet, this choice is not straightforward and the outcome of the analysis may vary due to a trade‐off between data quality and volume. This problem is particularly recurrent for less‐studied groups with patchy sampling effort. The latitudinal pattern of mosses richness remains inconsistent across studies and these may emerge purely from sampling artefacts. Our main objective here is to assess the effect of different curation criteria on this spatial pattern in the Temperate Northern Hemisphere (above 20° latitude). We contrasted the geographical distribution of moss species records and the latitude‐species richness relation obtained under different data curation scenarios. These scenarios comprehend five sources of taxonomical standardisations and eight data cleaning filters. The analyses are based on the selection of well‐surveyed cells at 100 km cell resolution. The application of some ‘data curation scenarios’ severely affects the number of records selected for analysis and substantially changes the proportion of richness per cell. The sensitivity to data curation becomes detectable at regional and at the cell scales showing a large shift in the latitudinal richness peak in Europe, from 60° N to 45° N latitude, when only preserved specimens are selected and duplicates based on date of collection and coordinates are excluded. Our results stress the importance of justifying the criteria used for filtering biodiversity data retrieved from biodiversity databases to avoid detecting misleading patterns. Curating records under particular criteria compromises the information in some areas displaying different spatial information of mosses. This problem can be ameliorated if data filtering is combined with identifying well‐surveyed cells, render relatively constant results under different combinations of filtering even for less well‐known groups such as mosses.

## Graphical abstract

To overcome any biodiversity data limitation and establish robust geographic patterns, ecologists have to curate records. However, the application of some ‘data curation scenarios’ may severely affect the number of records selected for analysis and substantially changes the proportion of richness per cell. This study stresses the importance of justifying the criteria used for filtering biodiversity data retrieved from biodiversity databases to avoid detecting misleading patterns.

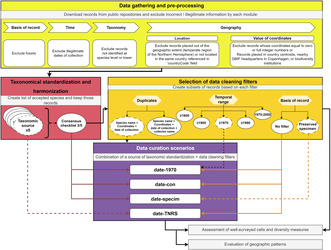

## 1 INTRODUCTION

Large‐scale biodiversity patterns are strongly determined by the quality and quantity of information available in species occurrences databases (Bisby, 2000; Hampton et al., 2013; Hughes, Orr, Yang, & Qiao, 2021; Moudry & Devillers, 2020). However, errors and inaccuracies in biodiversity information can generate artefacts and biases, which limit or mislead our understanding of the species distributions and perceived diversity patterns (Hawkins & Diniz‐Filho, 2008; Hortal et al., 2015; Hughes, Orr, Ma, et al., 2021; Maldonado et al., 2015).

Some challenges come from the dynamic nature of the species nomenclature which usually entails an overdescription in highly surveyed areas, different taxonomic treatments between regions or alternative spellings between databases (Feng et al., 2022; Isaac et al., 2004; Jansen & Dengler, 2010). Moreover, changes in species taxonomy due to splitting or lumping through time or unresolved taxa challenge the quantification of diversity measures (Jones et al., 2012; Stropp et al., 2022). Also, spatial biases in sampling effort cause an unevenly clustered distribution of occurrences along the world (Hortal et al., 2007; Hughes, Orr, Yang, & Qiao, 2021; Ladle & Hortal, 2013; Meyer et al., 2016; Oliveira et al., 2016; Troudet et al., 2017). Analyses of inventory completeness and ignorance scores can help determining the reliability of the knowledge and its geographical distribution (Ladle & Hortal, 2013; Rocchini et al., 2011; Sousa‐Baena et al., 2014; Stropp et al., 2016).

Researchers are required to first assess the strengths and limitations of data before establishing biodiversity patterns. For this, best practices in data management should include evaluating whether and to what extent the available biodiversity records are appropriate for their purposes (Gueta & Carmel, 2016; König et al., 2019; Vandepitte et al., 2015). In the context of biogeography, it is essential to check the accuracy of the geographical, taxonomical and temporal information in biodiversity records (Chapman, 2005; Meyer et al., 2016). This can be performed by using one of the numerous tools dedicated to the curation of species occurrence records (e.g. Jin & Yang, 2020; Ribeiro et al., 2022; Verborgh & De Wilde, 2013; Zizka et al., 2019). However, this raises the question of how different criteria for taxonomic standardisation and data cleaning filters may impact observed biogeographical patterns. Consequently, biodiversity metrics (e.g. richness) and their inferred large‐scale patterns could be inconsistent and distorted across space and time (Hughes, Orr, Ma, et al., 2021; Hughes, Orr, Yang, & Qiao, 2021; Maldonado et al., 2015). However, the extent to which the selection of data based on different aspects of record quality may alter the perceived richness patterns of many taxa groups remains unassessed (Meyer et al., 2016; Zizka et al., 2020).

In this study, we focus on mosses (Bryopsida), a relatively poorly studied group of non‐vascular plants that has been unevenly sampled across regions (Chen et al., 2015; Cornwell et al., 2019; Ronquillo et al., 2020). Bryophyte taxa are under‐represented in public repositories (Meyer et al., 2016) and the information on their regional distributions is uneven (Mutke & Geffert, 2010) with notable gaps in tropical areas, south‐central Asia and Africa (Geffert et al., 2013; Magill, 2010). Worldwide, many regions remain poorly sampled and nearly all studies that have analysed diversity in mosses at large geographical scales have highlighted the lack of sufficient information (see, for example, Geffert et al., 2013; Magill, 2010). These shortcomings probably result in a lack of consistent results of their global biogeographic patterns and contradictory outcomes between different studies (Chen et al., 2015; Geffert et al., 2013; Mateo et al., 2016; Möls et al., 2013; Sanbonmatsu & Spalink, 2022; Shaw et al., 2005). Global studies show that tropical and extra‐tropical areas harbour comparable species richness suggesting there is no latitudinal diversity gradient for mosses (Geffert et al., 2013; Möls et al., 2013). However, regional analyses show a different picture. A weak but negative latitudinal diversity gradient has been found in China (Chen et al., 2015) and America (Shaw et al., 2005). Remarkably, in other regions such as Europe and South‐western South America, researchers found an inverse latitudinal gradient (Mateo et al., 2016; Rozzi et al., 2008).

Besides, it is frequent among diversity studies in mosses to use political boundaries as operational geographic units due to the lack of more precise data (see Chen et al., 2015; Shaw et al., 2005). This approach has known limitations (Hughes, Orr, Yang, & Qiao, 2021), but to which extent it has biased our perception of the latitudinal diversity gradient is unknown. Moreover, mosses' taxonomy is under revision without an authoritative source at the global level, and recent works report more synonymizations than new species, suggesting an inflated number of valid species (Magill, 2010).

Our main objective is to assess the impact of different criteria for taxonomic standardisation and data filtering on our ability to describe accurately the geographical patterns of species richness. To do this, we apply a series of filtering and standardising methods to the moss records available in online databases across the temperate Northern Hemisphere. Specifically, we address four questions: (1) To what extent do different baseline data used for standardising species names affect the observed spatial patterns of species richness?; (2) To what extent do the geographical distribution of the number of records and species change using different criteria for filtering by (i) duplicate records, (ii) basis of records (i.e. field observation or existence of herbarium voucher) and (iii) temporal coverage; (3) To what extent do different filtering criteria affect the spatial coverage of well‐sampled cells?; (4) How do data curation scenarios affect the observed latitudinal gradient of richness at both regional and across study area?

## 2 METHODS

### 2.1 Data gathering and pre‐processing

We obtained moss occurrence records from four public repositories of biodiversity data including Global Biodiversity Information Facility (GBIF.org, 2021, https://www.gbif.org/), Consortium of North American Bryophyte Herbaria (CNABH, 2022), Botanical Information and Ecology Network (BIEN, Enquist et al., 2016; Maitner, 2022) and Integrated Digitised Biocollections (iDigBio, 2022). We discarded records from GBIF included in BIEN's repository because they were duplicates from GBIF dataset (Feng et al., 2022).

**FIGURE 1:**
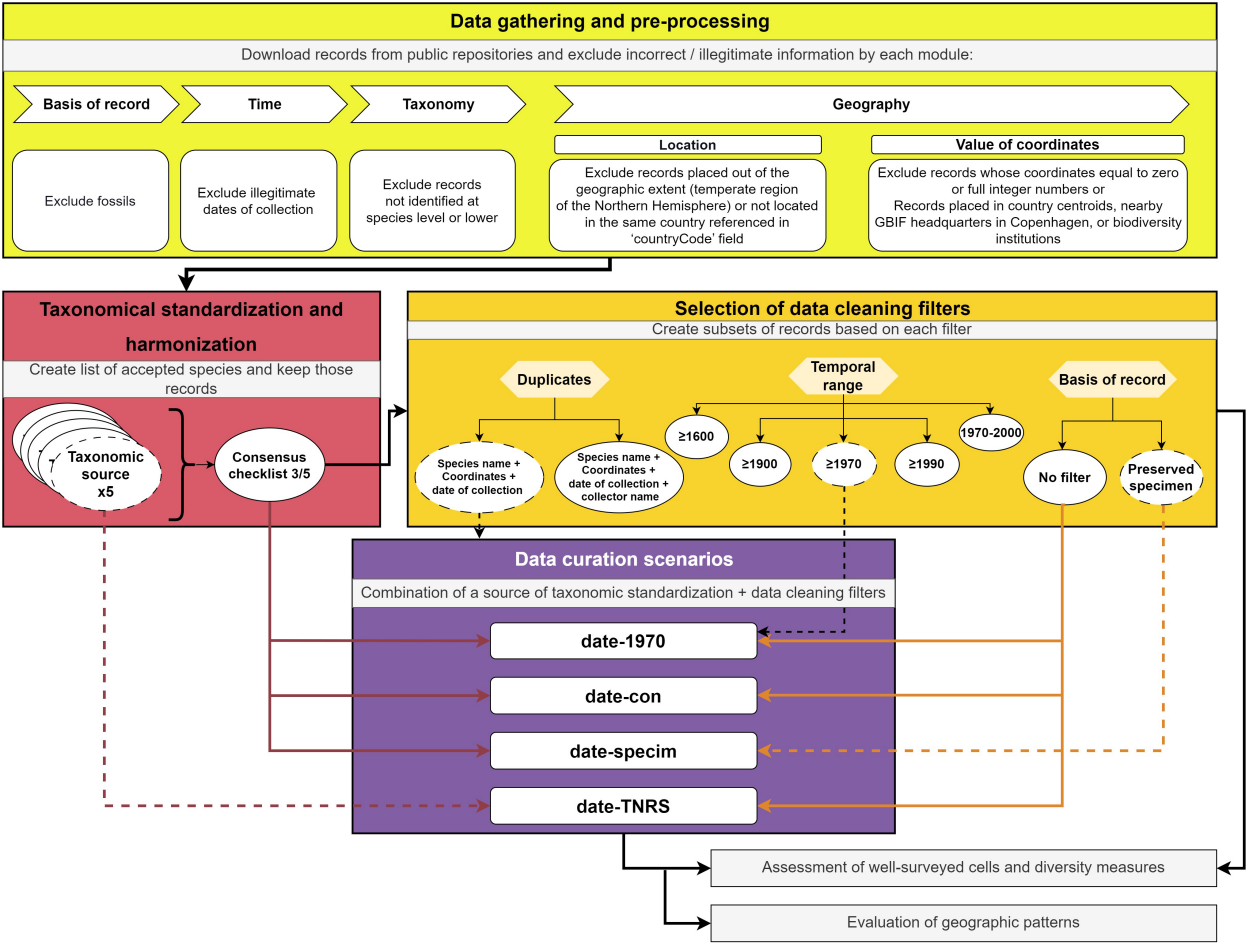
Workflow of the steps followed to process occurrence records. Coloured boxes correspond to the subsections described in the methods chapter. All the ‘data curation scenarios’ used the combination of species name, coordinates and date of collection to detect duplicates.

We retrieved only records from the temperate region of the Northern Hemisphere (above 20° N latitude, see Appendix Figure S1). This decision responded to the large differences in survey effort in Southern Hemisphere and tropical areas. We then followed the data processing protocol reviewed by OCCUR R Shiny App (C. Ronquillo, J. Stropp & J. Hortal, unpublished) to standardise and filter occurrence records used in the study, following different options at key steps (see below). The whole data management process was organised in a workflow connecting all the subsections of the methodology (Figure 1).

We pre‐processed our downloaded datasets individually to exclude fossil records, those with illegitimate dates of collection and those with the exact same value in their latitude and longitude coordinates. We also excluded records with geographic coordinates equal to zero and those given as full integer numbers (i.e. no decimal digits). We then retained those records placed in the same country referenced in ‘countryCode’ field (including a 0.1°, i.e. ~10 km, buffer from the nearest coastal point) or nearby the country border. Finally, we discarded records placed in country centroids, nearby GBIF headquarters in Copenhagen or biodiversity institutions (Zizka et al., 2019). We kept records identified at species level or lower (forms, subspecies and varieties).

Records were reprojected and aggregated to their overlapping cell on a grid of equal area cells of 100 km resolution (WGS 84/NSIDC EASE‐Grid 2.0 Global projection) covering our study region. The area was divided into three regions corresponding to North America, Europe and North Africa (herein often called simply Europe/N. Africa for short) and Asia. We generated 49 latitudinal bands dissolving each row from the grid (hereby numbered from ‘1’ to ‘49’ towards north) (see Appendix Figure S1).

All analyses and figures were made in RStudio (RStudio Team, 2021, version 4.1.0) using ‘data. table’ (Dowle & Srinivasan, 2021), ‘tidyverse’ (Wickham et al., 2019), ‘GADMTools’ (Decorps, 2021), ‘CoordinateCleaner’ (Zizka et al., 2019), ‘rnaturalearth’ (South, 2017), ‘lubridate’ (Grolemund & Wickham, 2011), ‘countrycode’ (Arel‐Bundock et al., 2018), ‘patchwork’ (Pedersen, 2020), ‘segmented’ (Muggeo, 2008), ‘KnowBR’ (Lobo et al., 2018), ‘rgbif’ (Chamberlain et al., 2022), ‘Taxondstand’ (Cayuela et al., 2021) and ‘WorldFlora’ (Kindt, 2020).

### 2.2 Taxonomical standardisation and harmonisation

After pre‐processing the records, we extracted a list of unique scientific names (dwc:scientificName and also dwc:species for iDigBio records). We standardised all names using five commonly used taxonomic databases for nomenclatural standardisation and harmonisation of plants (Grenié et al., 2022): GBIF backbone (using ‘rgbif’ R package), The Plant List (using ‘TaxonStand’ R package, http://www.theplantlist.org/), World Flora Online (using ‘WorldFlora’ R package, https://www.worldfloraonline.org/), Missouri Botanical Garden's Tropicos database (Tropicos; https://www.tropicos.org/) using TNRS web application setting ‘Tropicos’ option and Taxonomic Name Resolution Service (TNRS; Boyle et al., 2021) using TNRS web application with default settings (https://tnrs.biendata.org/). This procedure resulted in five different lists of accepted species names (shortened to only genus and specific epithet). We created a consensus species list from them as a conservative way to avoid taxonomic conflicts between those lists. Only those species names presented in at least three out of the five lists were selected generating the ‘taxConsensus’ dataset.

After excluding all species names that were marked as ‘unresolved’ or yielded no results (and their corresponding records), we quantified the proportion of species names observed by cell for each of the five taxonomic databases compared with the consensus list. We also calculated for each taxonomic database and region the proportion of cells for which the number of species names decrease, increase or remain unchanged compared with the consensus list.

### 2.3 Selection of data cleaning filters and quantification of records

We selected eight filters generating the corresponding subsets from the ‘taxConsensus’ dataset. These filters have been used previously in ecology studies (Table 1). They defined two types of duplicate criteria, one type of specific nature of the record (i.e. basis of records) and five different temporal ranges. We then quantified the proportion of number of records and observed species discarded by cell after applying each filter separately.

**TABLE 1:** Data cleaning filters used with examples of references in which were used and ‘data curation scenarios’ selected from the combination of data cleaning filters and taxonomical standardisation.

(i) Duplicates	Species name + coordinates + date of collection (DD/MM/YYYY) + collector or observer name (dwc:recordedBy)	Monsarrat et al. (2019)
	Species name + coordinates + date of collection (DD/MM/YYYY)	Menegotto and Rangel (2018)
(ii) basisOfRecord	Preserved Specimen	Stropp et al. (2016)
(iii) Temporal	Record includes year of collection (≥1600)	Ribeiro et al. (2022)
	≥1900	Gueta and Carmel (2016)
	≥1970	*‘TempModerate’* Meyer et al. (2016)
	1970–2000	WorldClim variables' temporal range
	≥1990	Troia and McManamay (2016)
(iv) Data curation scenarios	date‐TNRS: Remove duplicates with identical date of collection using TNRS taxonomic standardisation	
	date‐cons: Remove duplicates with identical date of collection using a taxonomic consensus species list	
	date‐specim: Extract only records associated to preserved specimens and remove duplicate records with identical date of collection	
	date‐1970: Extract only records collected after 1970 and remove duplicate records with identical date of collection	

### 2.4 Mapping well‐sampled cells

We calculated the inventory completeness with the ‘KnowBR’ package (Lobo et al., 2018) for each cell considering each of the eight filtered datasets described in the previous section. For this, we established species accumulation curves (SACs) using the number of records and species documented in each spatial unit. The number of records would be positively related to survey effort so it is assumed as a proxy (Lobo et al., 2018). SACs were established by using the method ‘exact’ and ‘rational’ and we estimated the percentage of inventory completeness keeping the last part of the slope. Next, we defined well‐sampled cells as those with at least 100 records, inventory completeness ≥70%, records/richness ratio ≥ 5 and slope ≤ 0.1 (i.e. one new species recorded every 10 new records at the end of the species accumulation curve), after Lobo et al. (2018). Finally, we checked the distribution of the obtained values of ratio and slope compared to their completeness by cell (see Appendix Figure S2).

### 2.5 Assessment of the latitudinal gradient of species richness

We created four different ‘data curation scenarios’ from the combination of some of the eight filters and taxonomic databases of the previous sections to evaluate the effect on the documented latitudinal gradients of species richness (Figure 1). The selection was based on those filters that presented higher differences from the initial dataset (see Appendix Table S1): (1) ‘date‐TNRS’: Remove duplicates with an identical date of collection using TNRS default system standardisation; (2) ‘date‐cons’: Remove duplicate records with identical date of collection using a taxonomic consensus species list; (3) ‘date‐specim’: Extract only records associated to preserved specimens and remove duplicate records with identical date of collection; (4) ‘date‐1970’: Extract only records collected after 1970 and remove duplicate records with identical date of collection. We then selected well‐sampled cells (see section 2.4 of methods) and generated regression models of their frequency and observed and predicted richness by latitudinal band—globally and for each of the three defined regions (see Appendix Figure S1). The relationships between latitude and richness were assessed through piecewise linear regressions (‘segmented’ Muggeo, 2008), assuming that the breakpoints identified by these regressions define the latitudinal peak of maximum species richness in each dataset.

## 3 RESULTS

**FIGURE 2:**
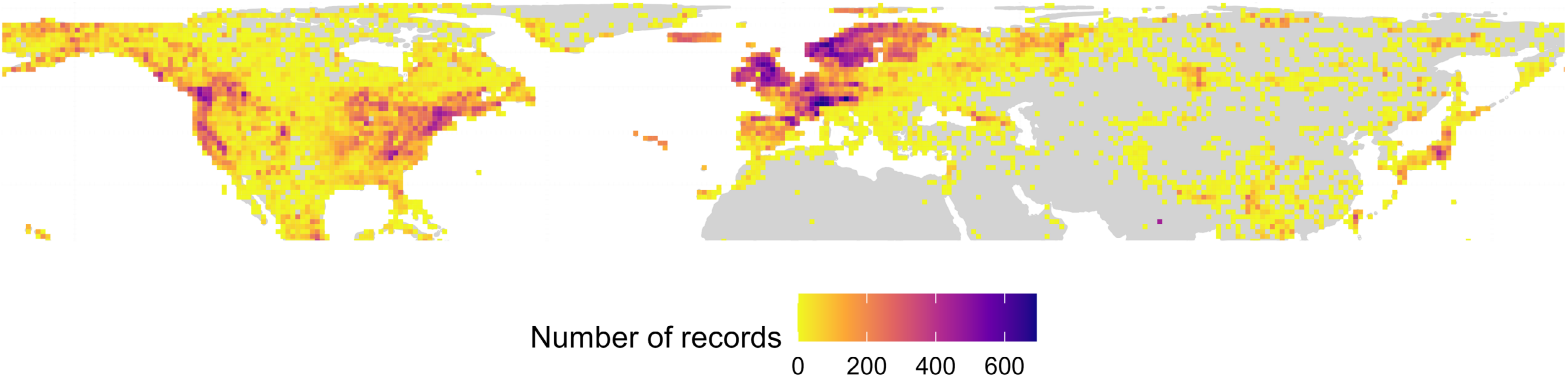
Geographical distribution of observed number of moss species in the ‘taxConsensus’ dataset at a 100 km grid cell resolution (NSIDC EASE‐Grid 2.0 Global projection).

After the pre‐processing steps, our dataset included 9,195,062 records attributed to 32,009 different taxa names, out of which 7000 corresponded to non‐standardised species names. These records were unevenly distributed across the regions: 22% in North America, 76% in Europe/N. Africa and 2% in Asia (Table S1). The dataset from GBIF had the largest number of records, with an initial number of ca. 11 million records, while data from CNABH comprises 1,539,928, from iDigBio 1,453,741 and from BIEN 1,273,086 records. After the taxonomic consensus processing, the number of records in the ‘taxConsensus’ dataset decreased to 9,069,440 records and it included 5693 different species (Figure 2).

### 3.1 Effect of standardising species names on observed spatial patterns of species richness

**FIGURE 3:**
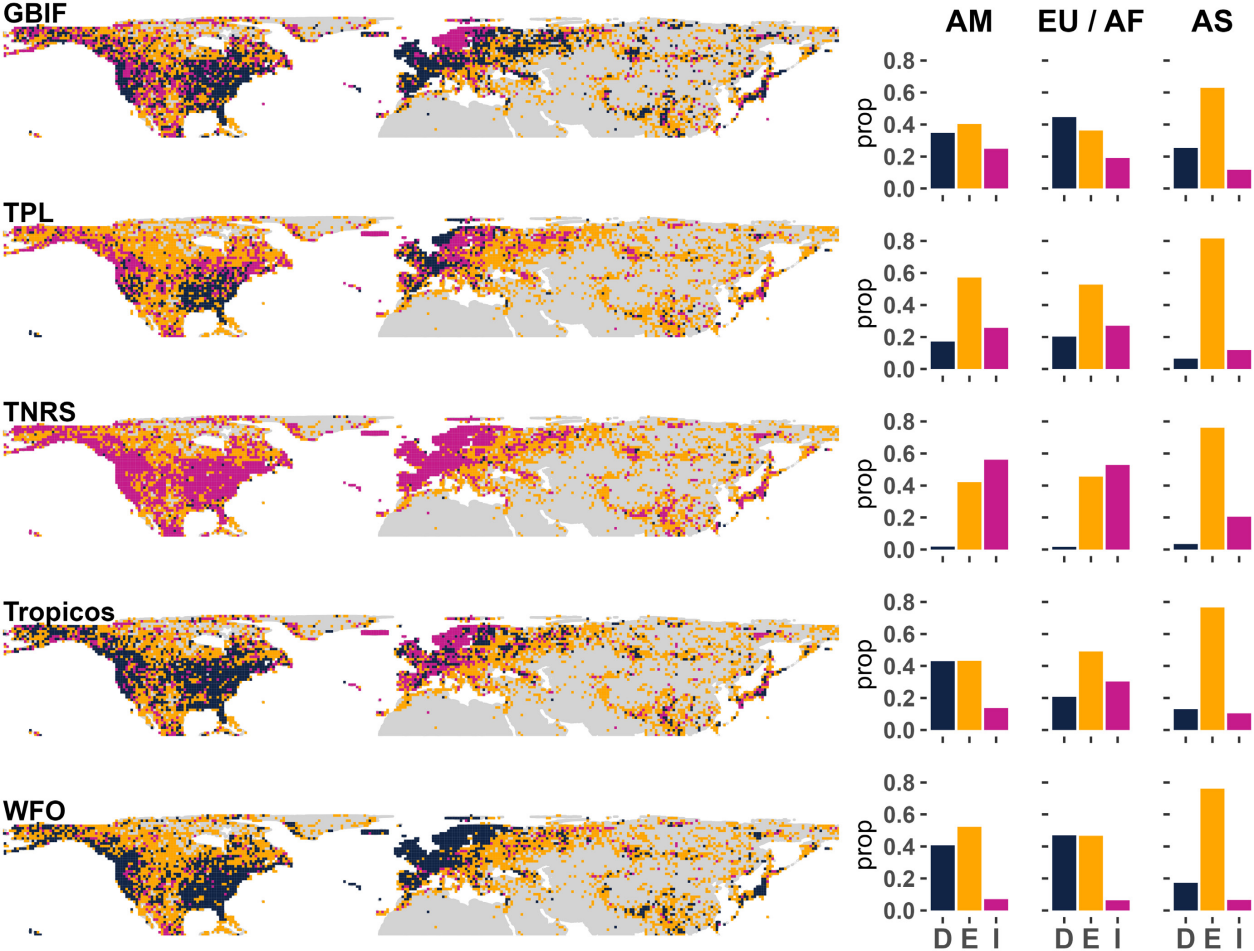
Geographical distribution of variations in number of observed species by cell at 100 km resolution (NSIDC EASE‐Grid 2.0 Global projection) using each taxonomic standardisation database: GBIF backbone, Taxonomic Name Resolution Service (TNRS), The Plant List (TPL), TROPICOS and WFO (World Flora Online) vs. a consensus checklist. Pink cells correspond to increases (I), blue to decreases (D) and yellow cells (E) are those with no variation in the number of observed species. Histograms represent the proportion of cells in each of these classes by region (AM = North America; EU/AF = Europe and North Africa; As = Asia).

The application of different taxonomic databases for standardisation led to different numbers of accepted species names: 5147 for GBIF backbone, 5636 for TPL, 6121 for TNRS, 5392 for Tropicos and 5611 for WFO and also number of records (see Table S1 to check each taxonomical source). There are also differences in the observed species richness by cell with a large proportion of cells that either increased or decreased their value compared to the use of a consensus checklist (Figure 3). However, most of these variations in observed richness represented a low proportion (<1%) of species discarded or added (see Appendix Figure S3). Cells with few species showed larger variations (more prone to additions or deletions). Across taxonomic databases, TPL resulted in the same richness values by cell as the consensus list in >50% of them, but the rest of the taxonomic databases had strong region‐dependent effects in perceived richness. Using Tropicos triggered a decrease in the number of species recorded in ~40% of cells of North America while generating increases in ~30% of the cells of Europe/N. Africa compared to the consensus checklist. Using the default TNRS setting system caused increases in the number of observed species in most of the cells in Europe (>40%) and in North America (~60%) (Figure 3). Remarkably, Asia kept most of the information regardless of the taxonomic database, and 60–80% of the cells keep the same number of species by cell than using a consensus checklist.

### 3.2 Effects of filtering for duplicate records, basis of records or temporal coverage on geographical distribution of records and observed species richness

Filtering duplicates led to an uneven geographical distribution of discarded records. North America showed the highest proportion of duplicate records by cell (See Appendix Figure S4). The two different criteria used for defining duplicates led to similar geographical patterns of number of records discarded. The inclusion of ‘recordedBy’ information and date of collection to identify duplicates led to a decrease in data of ~17%, whereas only considering date of collection led to ~20% (see Table S1).

**FIGURE 4:**
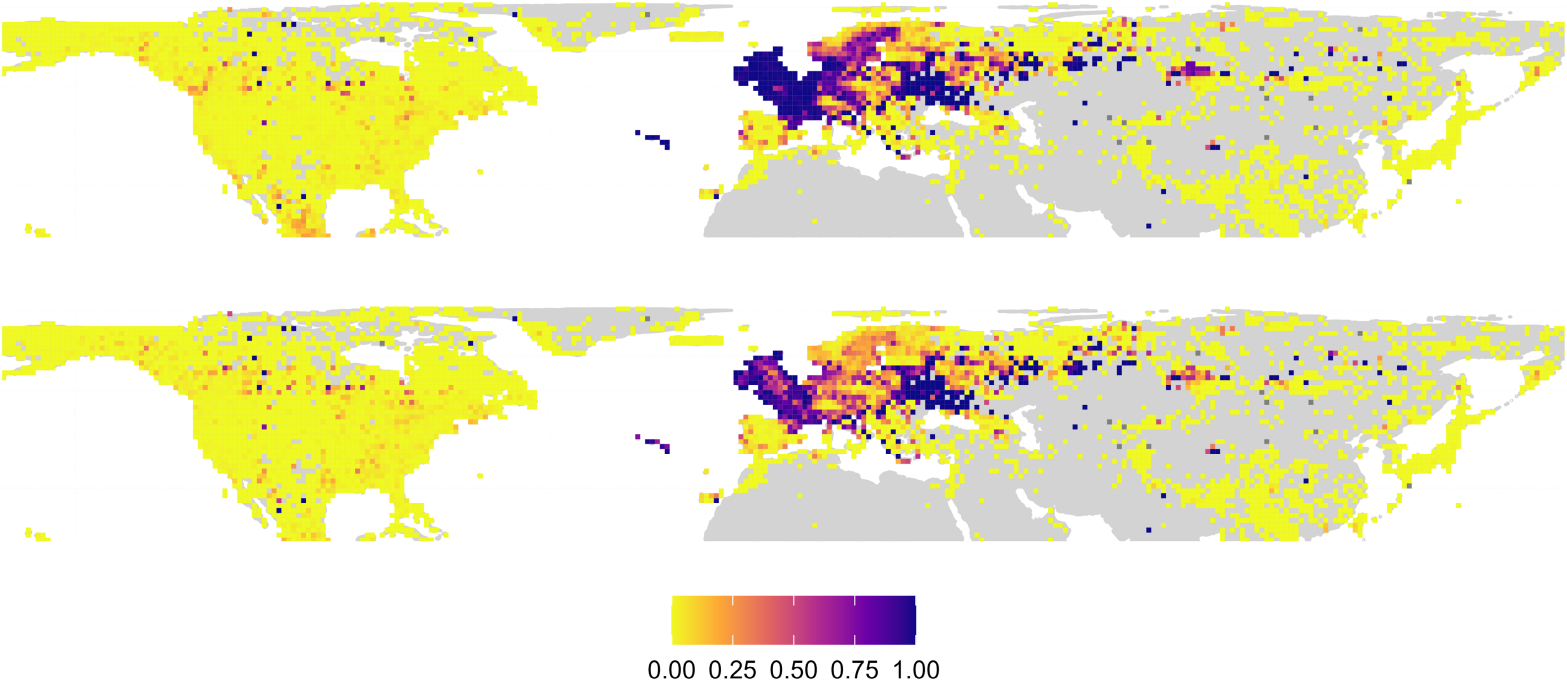
Geographical distribution of the proportions of records (top) and observed species (bottom) of mosses that were discarded after filtering to include only preserved specimens, at a 100 km cell width resolution (NSIDC EASE‐Grid 2.0 Global projection).

Data decreased unevenly by region when filtering by preserved specimens. Europe/N. Africa presents larger losses of records, which is also reflected in decreases in the number of observed species, of up to 80% by cell in areas of the UK, Azores, France and other central European countries (Figure 4).

**FIGURE 5:**
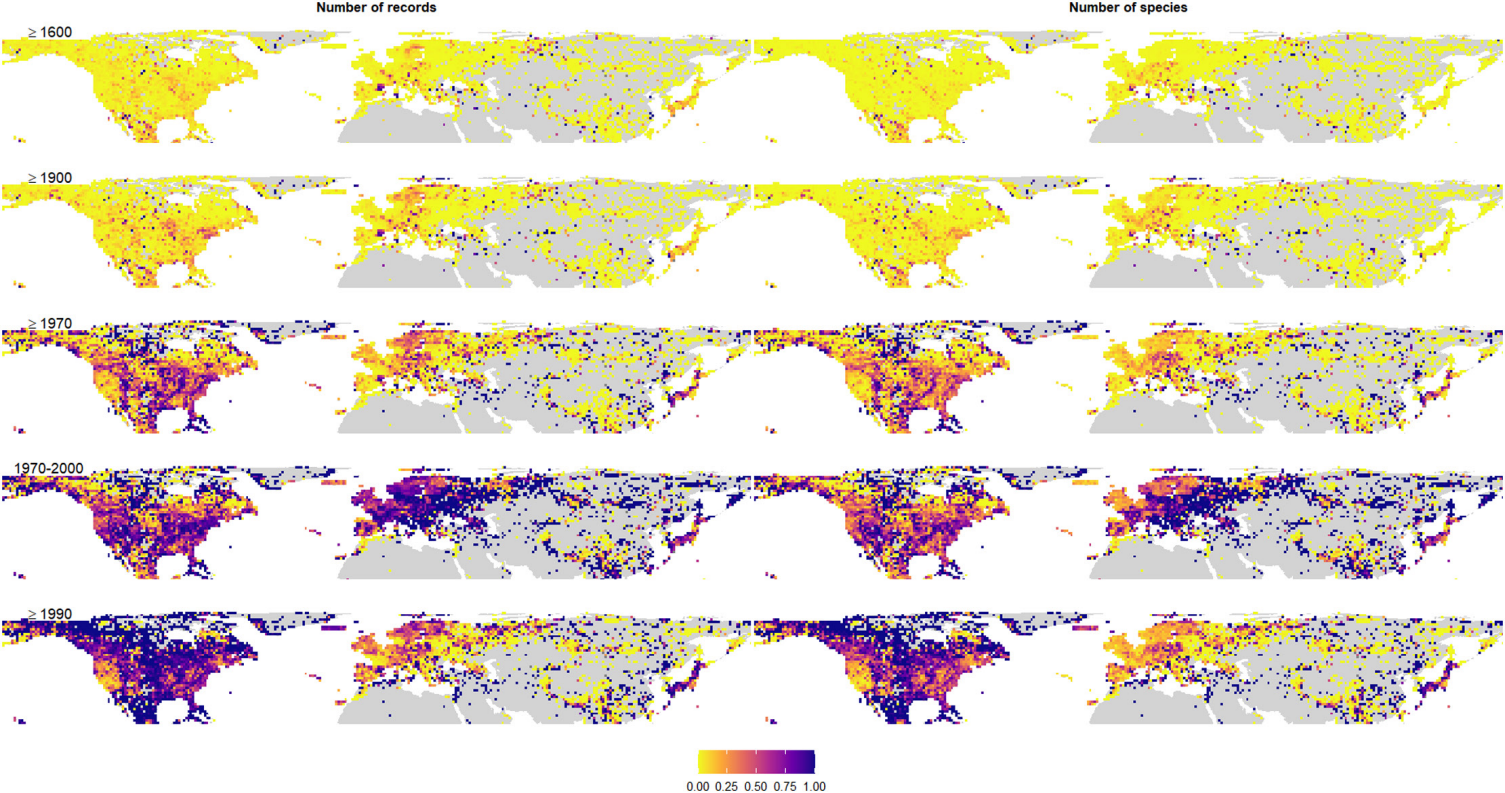
Geographical distribution of the proportions of records (left) and observed species (right) of mosses discarded after filtering by different temporal ranges, at 100 km cell width resolution (NSIDC EASE‐Grid 2.0 Global projection).

The records uploaded without date of collection comprised ca. 4% (Figure 5 Top). Further, the application of strict temporal filters, such as records collected between 1970 and 2000 or after 1990, reduced (up to 80%) the number of available records and species by cell throughout the whole study area (Figure 5). The North American region presented a higher loss of records in both time periods (1970–2000 and after 1990; Table S1). By contrast, in Europe/N. Africa this decrease is only pronounced between 1970 and 2000, as an important proportion of data was collected in the last two decades (Table S1).

### 3.3 Variation of the geographical distribution of well‐sampled cells according to data cleaning filters and ‘data curation scenarios’

**FIGURE 6:**
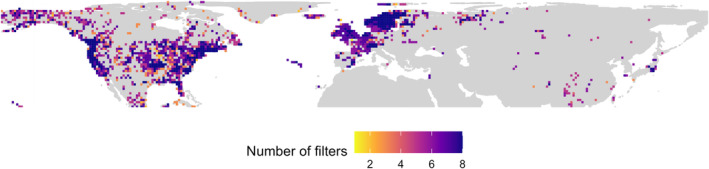
Geographic location of well‐sampled cells of mosses at 100 km cell width resolution (NSIDC EASE‐Grid 2.0 Global projection). Values correspond to number of filter criteria overcome of the eight proposed in Table 1.

We found that 1531 cells (out of 4969) can be considered well‐sampled after applying at least one of the eight cleaning filters proposed (Table 1) and 373 cells fulfilled all eight criteria (Figure 6). They were mostly located in Europe and North America (Figure 6; see Appendix Figure S5). However, cells across North America showed more variability than European ones (Figure 6) and some of them could not be considered well‐sampled after applying many of the filter criteria assessed (see Appendix Figure S5).

The numbers of well‐sampled cells of our four ‘data curating scenarios’ (Table 1) were: (1) ‘date‐TNRS’ 729 (371 in North America, 338 in Europe/N. Africa and 20 in Asia); (2) ‘date‐cons’ 967 (586 in North America, 359 in Europe/N. Africa and 22 in Asia); (3) ‘date‐specim’ 784 (578 in North America, 191 in Europe/N. Africa and 15 in Asia) and (4) ‘date‐1970’ 751 (388 in North America, 343 in Europe/N. Africa and 20 in Asia) (see Appendix Figure S5 for geographical distribution of cells).

### 3.4 Effect of ‘data curation scenarios’ on the observed latitudinal gradient of species richness

**FIGURE 7:**
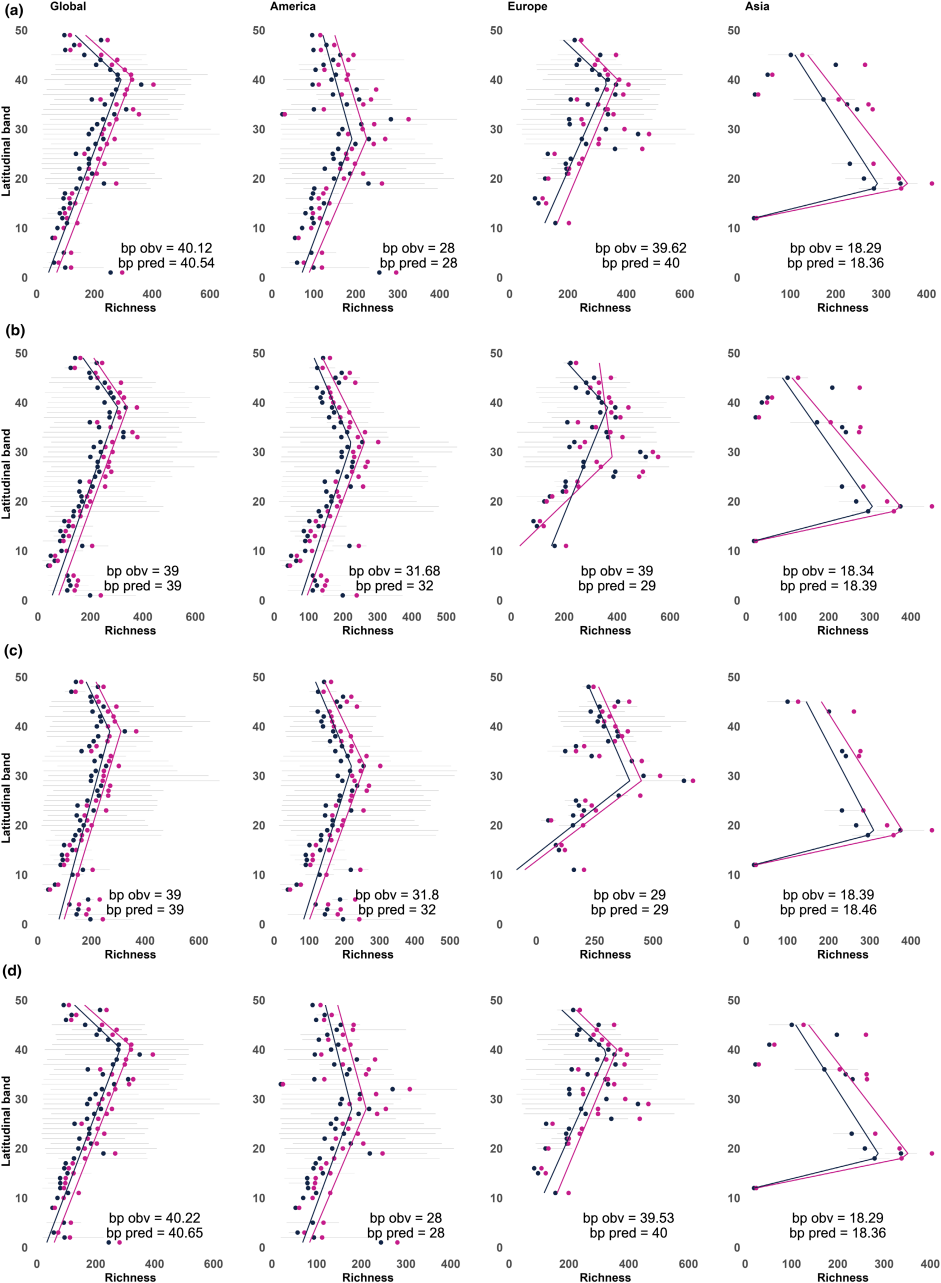
Global and regional latitudinal gradients of mosses species richness. Panels display ‘data curation scenario’: (a) date‐TNRS; (b) date‐cons; (c) date‐specim; (d) date‐1970. Dots depict median observed (blue) and predicted species richness (pink) and corresponding regression lines between these parameters and latitude are given in the same colour. Horizontal grey lines correspond to range of observed richness by latitudinal band. ‘Bp obs’ = breaking‐point values of piecewise regressions for observed richness and ‘bp pred’ = for predicted richness.

Regardless the criteria used to assess the latitudinal species richness gradient, we detected an increase in richness towards the north at global scale at ~60° N (latitudinal band ≈ 40) (Figure 7). Remarkably, the latitudinal coverage of data and peak in species richness are similar in all the curation scenarios. However, peaks of species richness differed by region; it was located also at ~60° N in Europe/N. Africa (latitudinal band ≈ 40), but shifted to ~45° N in North America (latitudinal band ≈ 30) and ~35° N in Asia (latitudinal band ≈ 18) (Figure 7). There were no differences between piecewise regressions of observed richness and predicted richness between ‘data curation scenarios’ except for ‘date‐cons’ in Europe/N. Africa (Figure 7b). After keeping only preserved specimens' records and deleting duplicates by date of collection, the peak of species richness shifts from ~60° N to ~45° N (latitudinal bands 40 and 29) (Figure 7c). All regression models present *R* ^2^ below .15 for the entire study area, below .10 for Europe/N. Africa and North America but above 0.5 for Asia due to the few points available in this region (see Appendix Tables S2–S5 for complete model summaries).

## 4 DISCUSSION

The criteria used to clean species occurrence records of mosses have a significant impact on the final data used in analyses and the associated results. Further, such impact may vary depending on the region or the temporal and spatial scale considered. Severe loss of information on the perceived species richness occurred at small scale, but it did not impact the large‐scale perception of latitudinal gradient considering well‐surveyed cells. The selection of source for taxonomic standardisation and data cleaning filters (e.g. temporal and basis of records) presented a trade‐off between getting suitable data and the limited number of records available with these characteristics. Our results showed that publicly available data on mosses is unevenly distributed across the temperate region of the Northern Hemisphere, a pattern that was also observed in previous studies with other groups (Hughes, Orr, Ma, et al., 2021). Indeed, well‐sampled cells are spatially biased towards northern (and high‐income) countries (Hughes, Orr, Ma, et al., 2021; Meyer et al., 2016).

We also showed that species occurrence records of mosses are geographically clustered by certain data types. This was also observed in Europe/N. Africa, which is the better‐sampled region in the Northern Hemisphere, with ~76% of the records of the global dataset. Our results revealed that filtering by preserved specimens is critical for data on mosses from a large part of Europe (e.g. the UK, France, Germany, Belgium or the Azores). In this region, most of the information was recorded as observations. Thus, selecting only preserved specimens shifted the peak of richness from ~60° N of latitude towards the south to ~45° N (Figure 7c). Filtering only preserved specimens has been used in previous works of ecology for other plant groups (Speed et al., 2018; Stropp et al., 2016). One of the reasons for keeping only records of preserved specimens could be the revision of sample material to validate species identifications. However, we showed that this kind of decision may be justified based on the extent and region or taxa chosen. Studies should give a clear motivation to exclude records with no specimen material associated or keep a conservative scenario with no filters applied.

Another crucial aspect to consider when filtering records is the selection of a particular temporal coverage. Data‐driven uncertainty increases as records get older (Meyer et al., 2016), due to issues of taxonomic validity, metadata quality and temporal changes in species composition (Stropp et al., 2022; Tessarolo et al., 2017). This calls for selecting only recent records for most analyses on current biodiversity trends due to their higher reliability (Tessarolo et al., 2017). However, our results showed that for mosses adjusting the information to a couple of decades before or after 1990 can produce larger decreases in both numbers of records and observed richness in Europe than in North America or vice‐versa (Figure 5). So, for the sake of obtaining datasets that are large enough to compare two regions with different histories of survey effort, it may be worth sacrificing temporal data quality to ensure large spatial coverage. Of course, bearing in mind the problems and limitations in the results derived from this choice. Nonetheless, the problem of temporal coverage may also be larger at progressively smaller extents of analysis. For example, while in this study records with no date of collection constituted only a small proportion (ca. 4%) of all the databases analysed, in a previous work focused in Iberian Peninsula more than 40% of moss records had no date information (Ronquillo et al., 2020). That renders these data unsuitable for analysis of temporal shifts in biodiversity. Indeed, this exemplifies the importance of recording high‐quality metadata when digitising biodiversity databases (Costello et al., 2013).

We found that filtering for duplicate records considering collectors' names had little impact on the number and spatial coverage of records of mosses. This is probably due to the lack of standardised information regarding collectors' names in global biodiversity data repositories. Ideally, databases should incorporate a systematised way of recording the collector or collectors of each record, and researchers or non‐professional users engage in filling it in. However, distinguishing occurrences collected by the same person on the same day and location from those collected by multiple collectors (and thus obtained from a higher level of sampling effort) is often challenging. Therefore, all the ‘data curation scenarios’ chosen for analysing the latitudinal gradients of richness filters records with the same date of collection, coordinates and species name.

We showed that observed species richness by region or cell is sensitive to the choice of the reference baseline data by using five different sources of taxonomic standardisation. For example, we found that GBIF backbone presented a lower number of accepted species than the others, but generated higher values of observed richness in some areas (Figure 3). Each taxonomic database has its approach or strategy and instability (Feng et al., 2022). World Flora Online, Tropicos or The Plant List only describes plant taxonomy and the latest is outdated since 2015, while GBIF backbone describes all taxonomy. Additionally, there are differences in the system's efficiency to detect spelling variations and generate an output (e.g. using orthographic distance metrics) (Grenié et al., 2022). Taxonomical Name Resolution Service (TNRS) can be configured to consider multiple checklists (e.g. Tropicos, WFO, USDA) to standardise a species name. That could be the main reason why this taxonomic setting caused general increases of richness at cell level in all regions compared to the other sources assessed (Figure 3).

Expert knowledge is key when selecting the taxonomic reference for standardisation (arguably Tropicos for bryologists, Magill, 2010). However, we showed the importance of this selection and how it could modify richness information. This finding calls the attention of ecologists to carefully consider which baseline for taxonomic information should be considered when conducting studies at a global scale. Here, we created a ‘consensus list’ with accepted species names from the majority of available sources (three out of five in this study) as a potential solution to address conflicts in the absence of an authoritative taxonomic reference. This allowed us to work with more records and species than just using one source of taxonomic standardisation. It is still unknown how many species of mosses exist. Global evaluations date back to the last decade and range between ca. 9000 (Magill, 2010) and ca. 13,000 (Crosby et al., 1999). In this study limited to the temperate region of the Northern Hemisphere, we captured a large proportion of the total species predicted by the experts (around 5500).

The limited information from Asia in public repositories, such as GBIF, highlight the difficulty to access records from this area due to language barriers. This region had a small number of available records, few cells with information and even fewer of them well‐sampled, even in countries with a large influence in bryology such as Japan or China (Chen et al., 2015). Consequently, it is less reliable to develop any biogeographical pattern for mosses in this region and to establish large‐scale comparisons. A massive mobilisation of data from Asian countries and its incorporation into international biodiversity information networks is needed.

Despite the uneven data coverage throughout the study area, our regional analyses provided evidence that there is not a single (unique) global latitudinal gradient of moss species richness. The latitudinal region exhibiting the highest richness peak of differed between North America, Europe/N. Africa and Asia. This peak, however, did not change significantly with the use of different ‘data curation scenarios’. In Asia the peak in species richness was placed further south compared to Europe or North America, although this observation should be interpreted with caution due to the limited number of cells included in the analysis. However, this result seems robust as it was maintained despite filter combinations and it was also consistent with previous analyses of the latitudinal biodiversity gradients in mosses (Chen et al., 2015; Mateo et al., 2016). We showed that the latitudinal diversity gradient in North America has a peak at intermediate latitudes. Differences in the latitudinal diversity gradient between regions are also known for vascular plants (Mutke & Barthlott, 2005), but the shift in the peak of species richness towards the North in Europe and the South in Asia (Mutke & Barthlott, 2005) is substantially larger in mosses.

Several hypotheses have been invoked to explain the differences in the latitudinal diversity gradient between mosses and vascular plants (see Patiño & Vanderpoorten, 2018). The environmental requirements (temperature and water availability) have been commonly advocated as responsible for the differences in the biodiversity patterns between both groups (Kreft & Jetz, 2007; O'Brien, 2006). However, there are intrinsic differences between vascular plants and mosses regarding optimum growth temperatures (Furness & Grime, 1982), and maintenance of water balance—as the lack of cuticles in mosses allows them to absorb water directly from mist and fog through their whole surface. Due to these particular characteristics, factors such as coastline length and air humidity based on the orography and levels of continentality show high correlations with moss richness (Möls et al., 2013) and may explain some of the differences in the latitudinal gradient between regions. However, historical contingencies have also played a significant role in shaping these differing patterns. Importantly, none of the basal clades in the moss lineage originated in the tropics, and the whole moss clade could have originated in the Southern Hemisphere (Shaw et al., 2005). These two contingencies may have also marked the migration and diversification mosses creating the differences in regional patterns that we observe today.

The limited knowledge of the origin of the regional differences in moss latitudinal gradient, compared with other groups, calls for a deeper exploration of the determinants of moss richness gradients. Previous literature is based on distributional models (Mateo et al., 2016) or countries' checklists (Geffert et al., 2013; Mutke & Geffert, 2010), but the lack of global high‐quality data makes it difficult to make definitive conclusions. Indeed, analyses of particular regions showed that it is crucial to consider the effects of survey effort on the perceived moss diversity gradients (e.g. China—Chen et al., 2015; Iberian Peninsula—Ronquillo et al., 2020). Our work considered the effect of survey effort using inventory completeness in a larger study area with millions of records. Well‐surveyed areas selected were quite stable between ‘data curation scenarios’ covering a substantial proportion of the study area. Despite of the limitations of public data for analysing large‐scale biogeographical patterns of poorly studied groups, we showed that most of the Northern Hemisphere has accumulated enough records of mosses to assess large‐scale patterns.

## 5 MAIN CONCLUSIONS

In this study we evaluated some of the most commonly used filters to clean records of species occurrences in macroecology and biogeography. It is widely known that selecting or excluding records based on different criteria can compromise the volume of data available. However, this may also compromise the reliability of the results, so the effect of different ways of filtering records needs to be assessed in works based on information from biodiversity databases. Here, we demonstrated that we could assess the robustness of macroecological patterns based on opportunistic records from public databases. Such assessment can be done using sensitivity analyses based on the combination of completeness analyses to detect well‐surveyed cells and basic macroecological analyses. Importantly, some combinations of filters can display different spatial information patterns.

(Macro)ecologists and biogeographers should consider public data limitations such as survey effort, inconsistent taxonomic information or quality of digitalization by different sets of researchers when modelling and evaluating diversity patterns. This implies that studies using biodiversity data records should detail and justify how they filter and standardise the taxonomy of species occurrences, as well as spatial data quality and accuracy (Rocchini et al., 2023; Zizka et al., 2020). There are a series of good practices in biodiversity data management that should be followed in similar studies but evaluating other taxa or regions. First, it is important to document the use of scripts and publish the code (either in R or in other languages) in open repositories such as GitHub, to make the whole process comparable and reproducible (Ivimey‐Cook et al., 2023). Second, researchers should review the most recent packages and web services to manage occurrence records and use the most updated source of taxonomy available (Grenié et al., 2022). Third, we recommend assessing the spatial coverage of survey effort across the study area to identify biases and data gaps caused by differences in regional histories of sampling (Hortal & Lobo, 2005; Ronquillo et al., 2020). And last, studies should consider including an appendix or supplementary material with a detailed section of the workflow and curation scenarios selected to process the data (C. Ronquillo, J. Stropp & J. Hortal, unpublished). In this respect, justifications for any decision that implies a large loss of information must be clarified in the text whenever possible.

The noise and artefacts coming from inadequate handling of data can alter the perceived spatial patterns of biodiversity (see Hortal et al., 2015; Tessarolo et al., 2021; Whittaker et al., 2005 for a review). Macroecology is necessarily a data‐intense science, and in the era of big data using all information at hand may result in analysing erroneous patterns. Conducting sound analyses requires addressing the trade‐off between data quality and data volume commonly presented in large public repositories of biodiversity information. Building up truly methodologically solid macroecology requires ways of incorporating this trade‐off into the biogeographer's toolbox.

## AUTHOR CONTRIBUTIONS

**Cristina Ronquillo:** Conceptualization (equal); data curation (lead); formal analysis (lead); methodology (lead); software (lead); visualization (lead); writing – original draft (lead). **Juliana Stropp:** Conceptualization (equal); formal analysis (supporting); supervision (equal); validation (supporting); visualization (supporting); writing – original draft (supporting); writing – review and editing (equal). **Nagore G. Medina:** Methodology (supporting); supervision (supporting); validation (supporting); writing – review and editing (equal). **Joaquin Hortal:** Conceptualization (equal); formal analysis (supporting); funding acquisition (lead); methodology (supporting); project administration (lead); supervision (supporting); validation (supporting); visualization (supporting); writing – original draft (supporting); writing – review and editing (equal).

## CONFLICT OF INTEREST STATEMENT

All authors declare that they have no conflicts of interest.

## Supporting information


Appendix S1.

## Data Availability

Data for this work comes from open sources listed in the reference section. The final combined and cleaned dataset ‘tempNorthBryo’ used for the analyses is available in digitalCSIC at doi.org/10.20350/digitalCSIC/14794, as well as the shapefiles. R Code is available on GitHub at https://github.com/cRonFer/bioDivRecordsAnalyses.
