# Supplementary material for: Exploring the impact of data curation criteria on the observed geographical distribution of mosses
Source: Ecol Evol. 2023 Dec 3;13(12):e10786. doi: 10.1002/ece3.10786 (PMC10694387; doi:10.1002/ece3.10786)
Supplement: Supplementary file 1 — Appendix S1. [file ECE3-13-e10786-s001.docx]

**Exploring the impact of data curation criteria on the observed geographical distribution of mosses**

**Cristina Ronquillo^1,2^, Juliana Stropp^1,3^, Nagore G. Medina^4,5^, Joaquin Hortal^1^**

1 Dept. Biogeography & Global Change, Museo Nacional de Ciencias Naturales (MNCN-CSIC), Madrid, Spain

2 Escuela Internacional de Doctorado, Universidad Rey Juan Carlos (URJC), Madrid, Spain

3 Department of Biogeography, Trier University, Trier, Germany

4 Dept. Biología (Botánica), Facultad de Ciencias, Universidad Autónoma de Madrid, Madrid, Spain

5 Centro de Investigación en Biodiversidad y Cambio Global (CIBC-UAM,) Facultad de Ciencias, Universidad Autónoma de Madrid, Madrid, Spain

**Correspondence:** Cristina Ronquillo, Dept. Biogeography & Global Change, Museo Nacional de Ciencias Naturales (MNCN-CSIC), Madrid, Spain. Email: cristinaronquilloferrero@gmail.com

**Appendix**

**
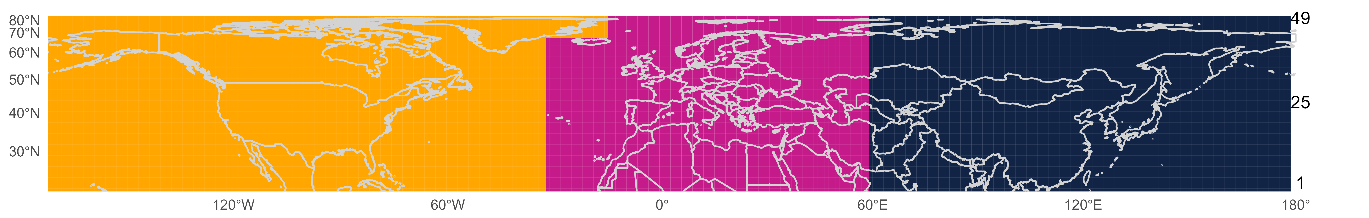
 FIGURE S1.** Study area regions considered in the analyses. North America (yellow); Europe + North Africa (pink) and Asia (blue). Second y-axis corresponds to latitudinal bands values.

**
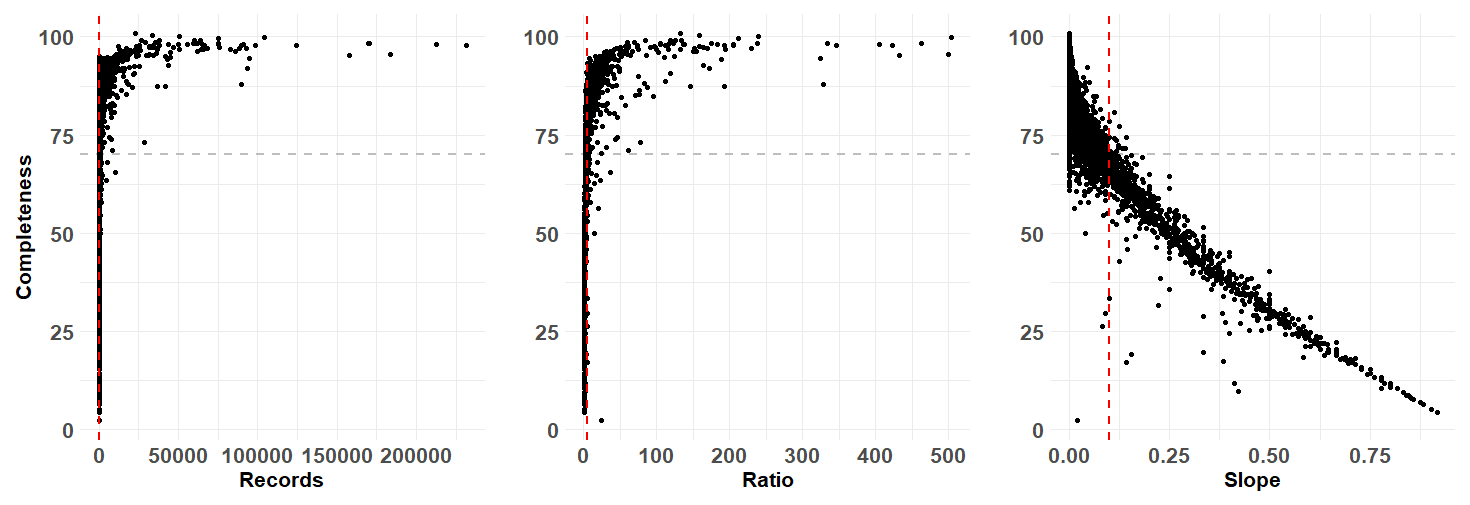
 FIGURE S2.** Distribution of records, ratio and slope values based on inventory completeness by cell. Red lines indicate selected thresholds criteria of well-sampled cells and horizontal grey line the completeness threshold.


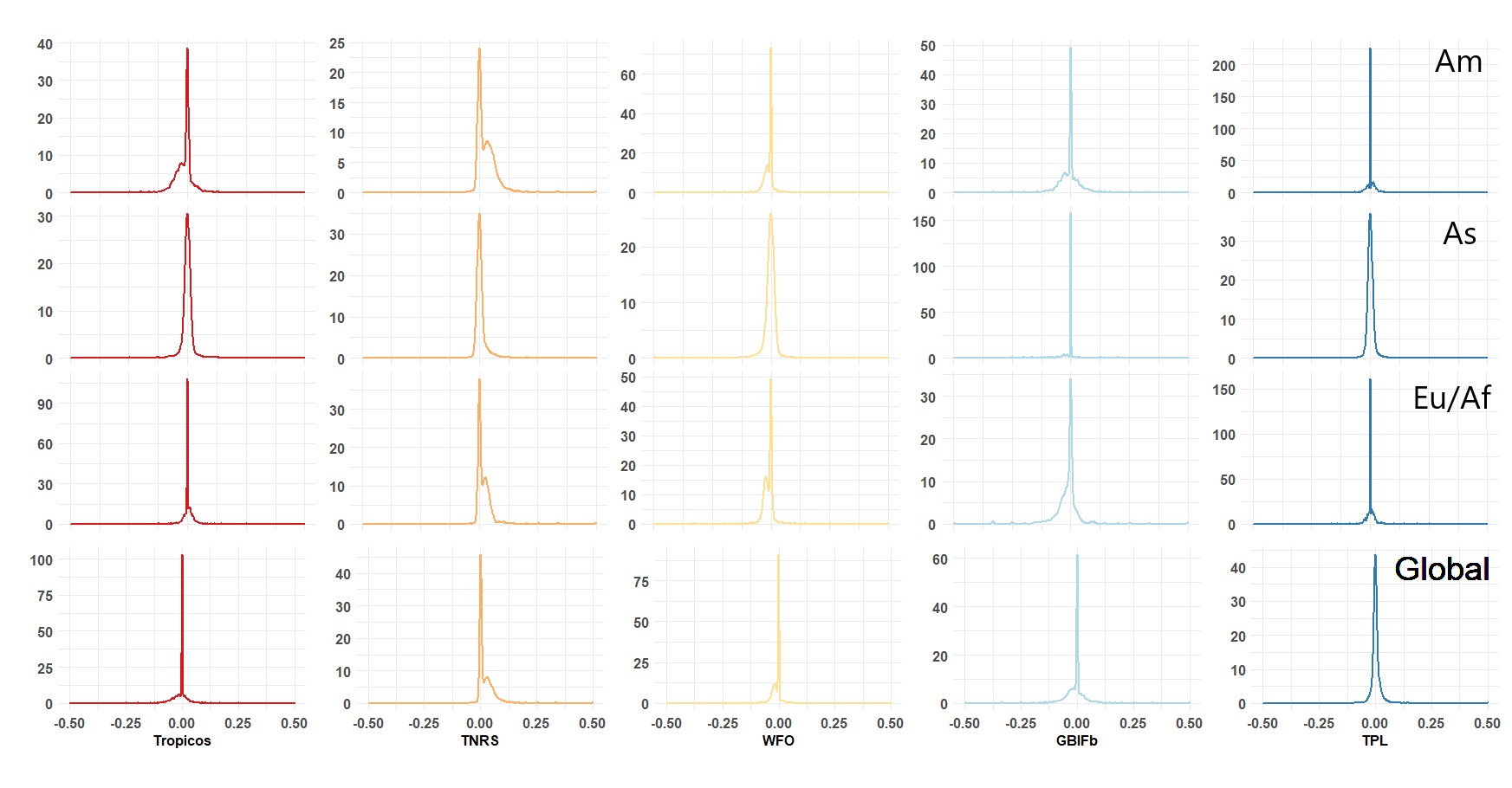
 **FIGURE S3.** Density of number of cells based on taxonomical richness change rates. 0 correspond to cells that keep the same number of species than using a consensus list, negative values are the proportion of species discarded in each cell compared to use a consensus checklist and positive values the proportion of species added. The x-range showed is limited to -0.5 - 0.5 for plot purposes. This was calculated for each taxonomic source assessed vs. a consensus checklist (columns: TNRS = Taxonomic Names Resolution Service; WFO = World Flora Online; GBIFb = Global Biodiversity Information Facility backbone and TPL = The Plant List) and region (rows: Am = North America; As = Asia and Eu/Af = Europe/ North Africa).


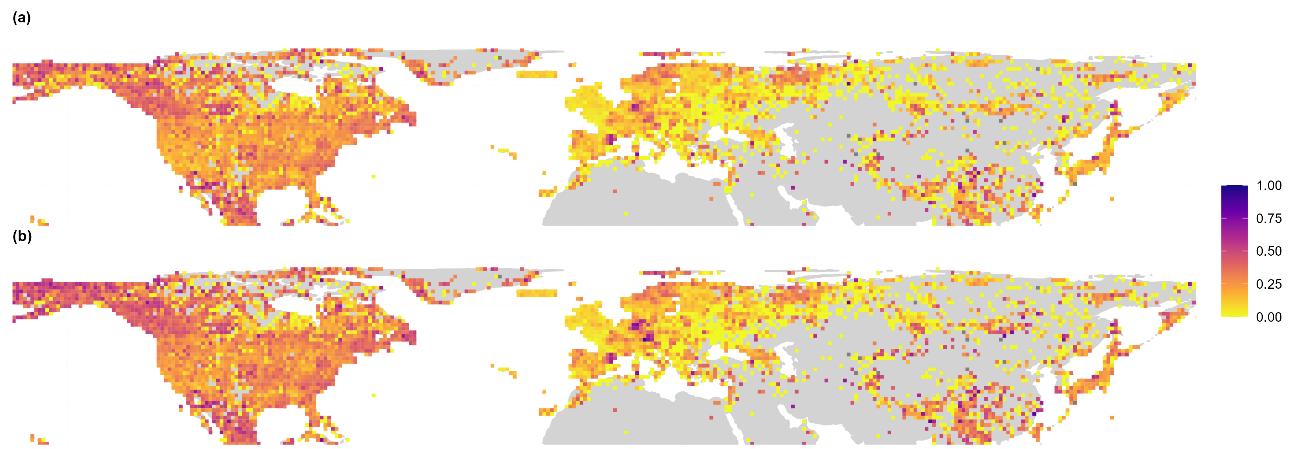
 **FIGURE S4.** Geographical distribution of the proportion of records discarded after remove duplicates with the same coordinates and date of collection (a) and same coordinates, ‘dwc:recordedBy’ collector’s names and date of collection (b) at 100 km resolution.

**
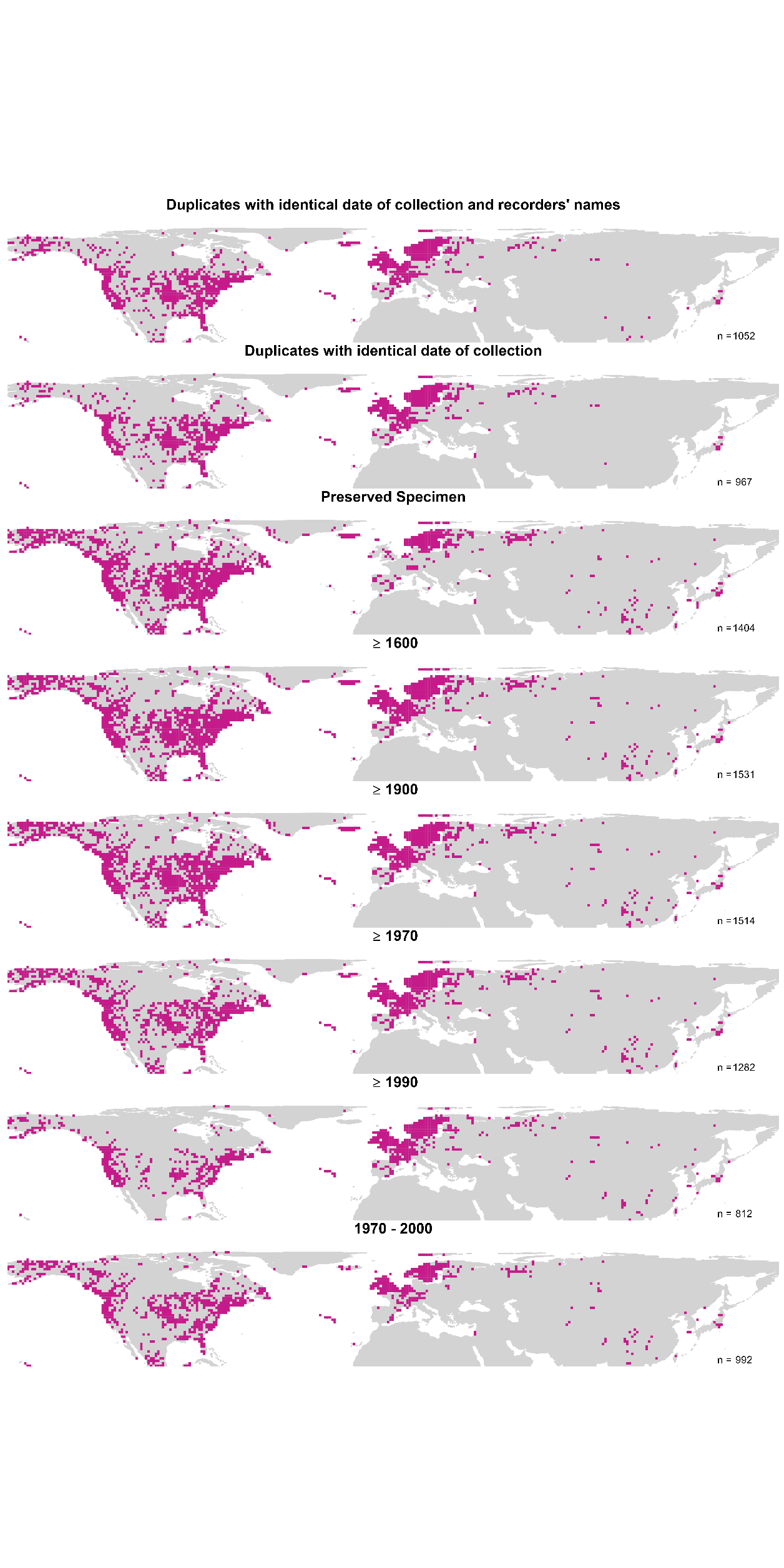
 FIGURE S5.** Geographic location of well-sampled cells based on each filter applied and ‘data curation scenario’ (n = number of cells).


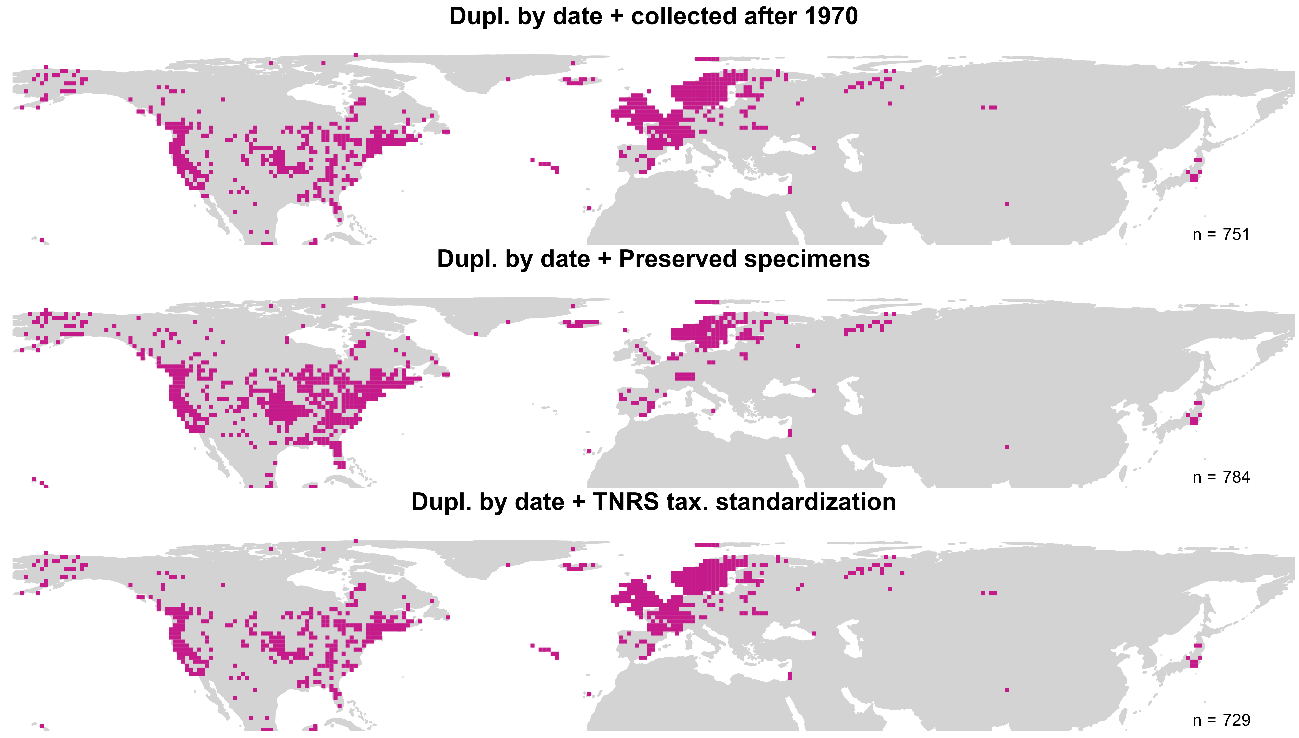
 **FIGURE S5. Continue**

| **TABLE S1**. Number and proportion of records retained after apply each data filter and ‘data curation scenario’ (combination of filters and taxonomical standardization). Proportion values are calculated based on number of records of the dataset with no filter. Values by region and global are included. Cells in red correspond to those that retained <25%; in orange 25-50% and yellow 50-75% | | | | | | | | | |
| --- | --- | --- | --- | --- | --- | --- | --- | --- | --- |
|  | | **Number of records** | | | | **Proportion of records** | | | |
|  |  | **North America** | **North Africa Europe** | **Asia** | **Global** | **North America** | **North Africa Europe** | **Asia** | **Global** |
| **(i) Duplicates** | **Species name + Coordinates + date of collection (DD/MM/YYYY) + collector or observer name (dwc:recordedBy)** | 1405210 | 5987996 | 113213 | 7506419 | **0.69** | 0.86 | **0.71** | 0.82 |
|  | **Species name + Coordinates + date of collection (DD/MM/YYYY)** | 1310406 | 5855304 | 106818 | 7272528 | **0.64** | 0.84 | **0.67** | 0.79 |
| **(ii) basisOfRecord: Preserved Specimen** | | 1955955 | 1196059 | 146822 | 3298836 | 0.95 | **0.17** | 0.93 | **0.36** |
| **(iii) Temporal** | **Record includes year of collection (>1600)** | 1903344 | 6656524 | 146735 | 8706603 | 0.93 | 0.95 | 0.93 | 0.95 |
|  | **> 1900** | 1789791 | 6425885 | 145349 | 8361025 | 0.87 | 0.92 | 0.92 | 0.91 |
|  | **> 1970** | 1147858 | 5608738 | 128517 | 6885113 | **0.56** | 0.80 | 0.81 | **0.75** |
|  | **1970 – 2000** | 765015 | 2331322 | 60821 | 3157158 | **0.37** | **0.33** | **0.38** | **0.34** |
|  | **> 1990** | 642467 | 4583397 | 98728 | 5324592 | **0.31** | **0.66** | **0.62** | **0.58** |
| **(iv) Data curation scenarios** | **date-TNRS:** | 1333894 | 5902843 | 107740 | 7344477 | **0.65** | 0.84 | **0.68** | 0.80 |
|  | **date-cons** | 1310406 | 5855304 | 106818 | 7272528 | **0.64** | 0.84 | **0.67** | 0.79 |
|  | **date-specim** | 1264890 | 875130 | 99880 | 2239900 | **0.62** | **0.13** | **0.63** | **0.24** |
|  | **date-1970** | 732448 | 4872738 | 89243 | 5694429 | **0.36** | **0.70** | **0.56** | **0.62** |
| **Taxonomical standards** | **GBIF** | 1995839 | 6598863 | 152736 | 8747438 | 0.97 | 0.94 | 0.96 | 0.95 |
|  | **TPL** | 2022379 | 6956622 | 156339 | 9135340 | 0.99 | 1.00 | 0.99 | 0.99 |
|  | **TROPICOS** | 2020080 | 6944446 | 155902 | 9120428 | 0.99 | 0.99 | 0.98 | 0.99 |
|  | **TNRS** | 2033774 | 6936521 | 156047 | 9126342 | 0.99 | 0.99 | 0.98 | 0.99 |
|  | **WFO** | 1985890 | 6822660 | 154194 | 8962744 | 0.97 | 0.98 | 0.97 | 0.97 |
| **Dataset with no filter** | | 2049626 | 6986879 | 158557 | **9195062** |  |  |  |  |

**TABLE S2.** Regression model summaries with segmented relationship(s) indicating estimated Break-Point (BP) of observed and predicted richness and frequency of well-sampled cells by latitudinal band at global scale and by region. ‘date-TNRS’ data curation scenario: data obtained after remove duplicates with identical date of collection and using TNRS standardization of species names.

| **Global Predicted Richness** | **Global Observed Richness** | **Global Frequency of cells** |
| --- | --- | --- |
| BP Est. St.Err | BP Est. St.Err | BP Est. St.Err |
| **psi1.latitudeBands 40.541 0.844** | **psi1.latitudeBands 40.12 0.9** | **psi1.latitudeBands 31.162 1.311** |
| Meaningful coefficients of the linear terms: | | |
| Estimate Std. Error t value Pr(>\|t\|) | Estimate Std. Error t value Pr(>\|t\|) | Estimate Std. Error t value Pr(>\|t\|) |
| (Intercept) 62.7526 19.0789 3.289 0.00105 ** | (Intercept) 34.8635 17.7488 1.964 0.0499 * | (Intercept) -7.1379 2.4639 -2.897 0.00602 ** |
| latitudeBands 6.6237 0.6506 10.181 < 2e-16 *** | latitudeBands 6.4333 0.6052 10.630 <2e-16 *** | latitudeBands 1.2462 0.1266 9.847 2.3e-12 *** |
| U1.latitudeBands -25.7792 6.1814 -4.170 NA | U1.latitudeBands -24.3901 5.7505 -4.241 NA | U1.latitudeBands -2.6415 0.2896 -9.121 NA |
| Signif. codes: 0 ‘***’ 0.001 ‘**’ 0.01 ‘*’ 0.05 ‘.’ 0.1 ‘ ’ 1 | | |
| Residual standard error 125.5 on 725 degrees of freedom | Residual standard error: 116.7 on 725 degrees of freedom | Residual standard error: 5.733 on 41 degrees of freedom |
| **R^2^:** **0.1418, Adj. R^2^: 0.1382** | **R^2^: 0.1486, Adj. R^2^: 0.1451** | **R^2^: 0.7619, Adj. R^2^: 0.7445** |
| Boot restarting based on 6 samples. Last fit: | | |
| Convergence attained in 5 iterations (rel. change 2.129e-13) | Convergence attained in 3 iterations (rel. change 1.9651e-11) | Convergence attained in 2 iterations (rel. change 7.9166e-1) |

| **America Predicted Richness** | **America Observed Richness** | **Frequency of cells in American region** |
| --- | --- | --- |
| BP Est. St.Err | BP Est. St.Err | BP Est. St.Err |
| **psi1.latitudeBands 28 1.953** | **psi1.latitudeBands 28 1.931** | **psi1.latitudeBands 27.146 1.375** |
| Meaningful coefficients of the linear terms: | | |
| Estimate Std. Error t value Pr(>\|t\|) | Estimate Std. Error t value Pr(>\|t\|) | Estimate Std. Error t value Pr(>\|t\|) |
| (Intercept) 83.915 24.677 3.401 0.000746 *** | (Intercept) 67.6946 21.5658 3.139 0.00183 ** | (Intercept) -2.9433 1.8894 -1.558 0.127 |
| latitudeBands 5.070 1.114 4.553 7.21e-06 *** | latitudeBands 4.3915 0.9732 4.512 8.64e-06 *** | latitudeBands 0.7804 0.1103 7.075 1.48e-08 *** |
| U1.latitudeBands -8.686 1.959 -4.434 NA | U1.latitudeBands -7.6773 1.7121 -4.484 NA | U1.latitudeBands -1.7372 0.1819 -9.548 NA |
| Signif. codes: 0 ‘***’ 0.001 ‘**’ 0.01 ‘*’ 0.05 ‘.’ 0.1 ‘ ’ 1 | | |
| Residual standard error: 102.2 on 367 degrees of freedom | Residual standard error: 89.28 on 367 degrees of freedom | Residual standard error: 4.069 on 40 degrees of freedom |
| **R^2^: 0.06759, Adj. R^2^: 0.05997** | **R^2^: 0.06723, Adj. R^2^:** **0.0596** | **R^2^: 0.7031, Adj. R^2^** : **0.6808** |
| Boot restarting based on 6 samples. Last fit: | Boot restarting based on 9 samples. Last fit: | Boot restarting based on 6 samples. Last fit: |
| Convergence attained in 2 iterations (rel. change 5.7481e-07) | Convergence attained in 1 iterations (rel. change 3.4205e-07) | Convergence attained in 2 iterations (rel. change 5.9635e-12) |

| **Europe Predicted Richness** | **Europe Observed Richness** | **Frequency of cells in European region** |
| --- | --- | --- |
| BP Est. St.Err | BP Est. St.Err | BP Est. St.Err |
| **psi1.latitudeBands 40 1.042** | **psi1.latitudeBands 39.619 0.99** | **psi1.latitudeBands 41.313 0.844** |
| Meaningful coefficients of the linear terms: | | |
| Estimate Std. Error t value Pr(>\|t\|) | Estimate Std. Error t value Pr(>\|t\|) | Estimate Std. Error t value Pr(>\|t\|) |
| (Intercept) 85.101 53.389 1.594 0.112 | (Intercept) 38.387 51.421 0.747 0.456 | (Intercept) -13.84255 2.65045 -5.223 1.87e-05 *** |
| latitudeBands 7.124 1.621 4.396 1.49e-05 *** | latitudeBands 7.494 1.561 4.801 2.38e-06 *** | latitudeBands 0.86825 0.08944 9.707 3.92e-10 *** |
| U1.latitudeBands -23.823 6.694 -3.559 NA | U1.latitudeBands -25.440 6.448 -3.946 NA | U1.latitudeBands -3.79278 0.78654 -4.822 NA |
| Signif. codes: 0 ‘***’ 0.001 ‘**’ 0.01 ‘*’ 0.05 ‘.’ 0.1 ‘ ’ 1 | | |
| Residual standard error: 130.2 on 334 degrees of freedom | Residual standard error: 125.4 on 334 degrees of freedom | Residual standard error: 3.598 on 26 degrees of freedom |
| **R^2^: 0.07417, Adj. R^2^: 0.06585** | **R^2^: 0.08583, Adj. R^2^: 0.07762** | **R^2^: 0.8083, Adj. R^2^: 0.7862** |
| Boot restarting based on 6 samples. Last fit: | Boot restarting based on 6 samples. Last fit: | Boot restarting based on 6 samples. Last fit: |
| Convergence attained in 2 iterations (rel. change 2.1815e-07) | Convergence attained in 4 iterations (rel. change 6.2059e-14) | Convergence attained in 2 iterations (rel. change 6.4503e-12) |

| **Asia Predicted Richness** | **Asia Observed Richness** | **Frequency of cells in Asian region** |
| --- | --- | --- |
| BP Est. St.Err | BP Est. St.Err | BP Est. St.Err |
| **psi1.latitudeBands 18.364 1.243** | **psi1.latitudeBands 18.288 1.197** | **NA** |
| Meaningful coefficients of the linear terms: | | |
| Estimate Std. Error t value Pr(>\|t\|) | Estimate Std. Error t value Pr(>\|t\|) | Estimate Std. Error t value Pr(>\|t\|) |
| (Intercept) -611.58 291.32 -2.099 0.0520 . | (Intercept) -508.00 234.37 -2.168 0.04562 * | (Intercept) 1.710563 0.590344 2.898 0.0159 * |
| latitudeBands 53.09 17.93 2.961 0.0092 ** | latitudeBands 44.00 14.42 3.050 0.00763 ** | latitudeBands -0.001451 0.018395 -0.079 0.9387 |
| U1.latitudeBands -61.53 18.07 -3.405 NA | U1.latitudeBands -50.98 14.54 -3.507 NA |  |
| Signif. codes: 0 ‘***’ 0.001 ‘**’ 0.01 ‘*’ 0.05 ‘.’ 0.1 ‘ ’ 1 | | |
| Residual standard error: 87.84 on 16 degrees of freedom | Residual standard error 70.66 on 16 degrees of freedom | Residual standard error 0.6829 on 10 degrees of freedom |
| **R^2^: 0.5887, Adj. R^2^** : **0.5116** | **R^2^: 0.6022, Adj. R^2^: 0.5276** | **R^2^: 0.0006219, Adj. R^2^: -0.09932** |
| Boot restarting based on 6 samples. Last fit: | | |
| Convergence attained in 2 iterations (rel. change 2.5139e-11) | Convergence attained in 2 iterations (rel. change 1.0527e-13) |  |

**TABLE S3.** Regression model summaries with segmented relationship(s) indicating estimated Break-Point (BP) of observed and predicted richness and frequency of well-sampled cells by latitudinal band at global scale and by region. ‘date-cons’ data curation scenario: data obtained after remove duplicates with identical date of collection using consensus taxonomic species list.

| **Global Predicted Richness** | **Global Observed Richness** | **Global Frequency of cells** |
| --- | --- | --- |
| BP Est. St.Err | BP Est. St.Err | BP Est. St.Err |
| **psi1.latitudeBands 39 1.099** | **psi1.latitudeBands 39 0.849** | **psi1.latitudeBands 29.822 1.311** |
| Meaningful coefficients of the linear terms: | | |
| Estimate Std. Error t value Pr(>\|t\|) | Estimate Std. Error t value Pr(>\|t\|) | Estimate Std. Error t value Pr(>\|t\|) |
| (Intercept) 72.748 16.559 4.393 1.24e-05 *** | (Intercept) 48.2585 16.0694 3.003 0.00274 ** | (Intercept) -6.9391 2.8646 -2.422 0.0196 * |
| latitudeBands 6.878 0.597 11.521 < 2e-16 *** | latitudeBands 6.5715 0.5917 11.106 < 2e-16 *** | latitudeBands 1.5741 0.1642 9.585 2.43e-12 *** |
| U1.latitudeBands -19.683 4.904 -4.014 NA | U1.latitudeBands -19.8238 3.8567 -5.140 NA | U1.latitudeBands -3.1967 0.3254 -9.823 NA |
| Signif. codes: 0 ‘***’ 0.001 ‘**’ 0.01 ‘*’ 0.05 ‘.’ 0.1 ‘ ’ 1 | | |
| Residual standard error: 134.1 on 963 degrees of freedom | Residual standard error: 125.9 on 963 degrees of freedom | Residual standard error: 7.245 on 44 degrees of freedom |
| **R^2^: 0.1372, Adj. R^2^: 0.1345** | **R^2^: 0.1405, Adj. R^2^: 0.1378** | **R^2^: 0.7562, Adj. R^2^: 0.7396** |
| Boot restarting based on 10 samples. Last fit: | Boot restarting based on 6 samples. Last fit: | Boot restarting based on 6 samples. Last fit: |
| Convergence attained in 1 iterations (rel. change 2.8913e-08) | Convergence attained in 5 iterations (rel. change 1.5876e-08) | Convergence attained in 3 iterations (rel. change 6.8423e-13) |

| **America Predicted Richness** | **America Observed Richness** | **Frequency of cells of American region** |
| --- | --- | --- |
| BP Est. St.Err | BP Est. St.Err | BP Est. St.Err |
| **psi1.latitudeBands 32 1.625** | **psi1.latitudeBands 31.677 1.715** | **psi1.latitudeBands 25.732 1.302** |
| Meaningful coefficients of the linear terms: | | |
| Estimate Std. Error t value Pr(>\|t\|) | Estimate Std. Error t value Pr(>\|t\|) | Estimate Std. Error t value Pr(>\|t\|) |
| (Intercept) 90.5093 17.9371 5.046 6.04e-07 *** | (Intercept) 74.8876 16.1501 4.637 4.37e-06 *** | (Intercept) -4.4272 2.4518 -1.806 0.078 |
| latitudeBands 5.3706 0.7681 6.992 7.45e-12 *** | latitudeBands 4.7087 0.6916 6.809 2.46e-11 *** | latitudeBands 1.2422 0.1621 7.662 1.43e-09 *** |
| U1.latitudeBands -12.5386 2.4236 -5.174 NA | U1.latitudeBands -10.9474 2.1821 -5.017 NA | U1.latitudeBands -2.5387 0.2408 -10.541 NA |
| Signif. codes: 0 ‘***’ 0.001 ‘**’ 0.01 ‘*’ 0.05 ‘.’ 0.1 ‘ ’ 1 | | |
| Residual standard error: 110.8 on 582 degrees of freedom | Residual standard error: 99.78 on 582 degrees of freedom | Residual standard error: 5.729 on 43 degrees of freedom |
| **R^2^: 0.0917, Adj. R^2^: 0.08702** | **R^2^: 0.08698, Adj. R^2^: 0.08228** | **R^2^: 0.7225, Adj. R^2^: 0.7031** |
| Boot restarting based on 7 samples. Last fit: | Boot restarting based on 6 samples. Last fit: | Boot restarting based on 6 samples. Last fit: |
| Convergence attained in 1 iterations (rel. change 3.3244e-08) | Convergence attained in 3 iterations (rel. change 8.0977e-13) | Convergence attained in 2 iterations (rel. change 6.7797e-12) |

| **Europe Predicted Richness** | **Europe Observed Richness** | **Frequency of cells of European region** |
| --- | --- | --- |
| BP Est. St.Err | BP Est. St.Err | BP Est. St.Err |
| **psi1.latitudeBands 29 1.719** | **psi1.latitudeBands 39 1.184** | **psi1.latitudeBands 42.202 0.744** |
| Meaningful coefficients of the linear terms: | | |
| Estimate Std. Error t value Pr(>\|t\|) | Estimate Std. Error t value Pr(>\|t\|) | Estimate Std. Error t value Pr(>\|t\|) |
| (Intercept) -180.695 134.183 -1.347 0.178957 | (Intercept) 68.687 54.571 1.259 0.209 | (Intercept) -14.21928 2.43772 -5.833 3.79e-06 *** |
| latitudeBands 19.371 5.511 3.515 0.000496 *** | latitudeBands 7.576 1.660 4.564 6.92e-06 *** | latitudeBands 0.89364 0.08071 11.072 2.44e-11 *** |
| U1.latitudeBands -21.912 5.775 -3.795 NA | (Intercept) 68.687 54.571 1.259 0.209 | U1.latitudeBands -4.46507 0.91696 -4.869 NA |
| Signif. codes: 0 ‘***’ 0.001 ‘**’ 0.01 ‘*’ 0.05 ‘.’ 0.1 ‘ ’ 1 | | |
| Residual standard error: 140.9 on 355 degrees of freedom | Residual standard error: 137.6 on 355 degrees of freedom | Residual standard error: 3.418 on 26 degrees of freedom |
| **R^2^: 0.06868, Adj. R^2^: 0.06081** | **R^2^: 0.07278, Adj. R^2^: 0.06495** | **R^2^: 0.8422, Adj. R^2^: 0.824** |
| Boot restarting based on 7 samples. Last fit: | Boot restarting based on 9 samples. Last fit: | Boot restarting based on 6 samples. Last fit: |
| Convergence attained in 4 iterations (rel. change 9.0925e-09) | Convergence attained in 2 iterations (rel. change -3.9343e-08) | Convergence attained in 2 iterations (rel. change 1.2113e-09) |

| **Asia Predicted Richness** | **Asia Observed Richness** | **Frequency of cells in Asian region** |
| --- | --- | --- |
| BP Est. St.Err | BP Est. St.Err | BP Est. St.Err |
| **psi1.latitudeBands 18.392 1.308** | **psi1.latitudeBands 18.341 1.27** | **NA** |
| Meaningful coefficients of the linear terms: | | |
| Estimate Std. Error t value Pr(>\|t\|) | Estimate Std. Error t value Pr(>\|t\|) | Estimate Std. Error t value Pr(>\|t\|) |
| (Intercept) -645.95 327.34 -1.973 0.0640 . | (Intercept) -535.00 264.72 -2.021 0.0584 | (Intercept) 1.67116 0.5634888 2.966 0.0128 * |
| latitudeBands 55.80 20.15 2.770 0.0126 * | latitudeBands 46.17 16.29 2.834 0.0110 * | latitudeBands 0.000682 0.01719 0.040 0.9691 |
| U1.latitudeBands -65.92 20.29 -3.248 NA | U1.latitudeBands -54.57 16.41 -3.325 NA |  |
| Signif. codes: 0 ‘***’ 0.001 ‘**’ 0.01 ‘*’ 0.05 ‘.’ 0.1 ‘ ’ 1 | | |
| Residual standard error: 98.7 on 18 degrees of freedom | Residual standard error: 79.82 on 18 degrees of freedom | Residual standard error: 0.6584 on 11 degrees of freedom |
| **R^2^: 0.5798, Adj. R^2^: 0.5098** | **R^2^: 0.5925, Adj. R^2^: 0.5246** | **R^2^: 0.000143, Adj. R^2^: -0.09075** |
| Boot restarting based on 6 samples. Last fit: | | |
| Convergence attained in 2 iterations (rel. change 5.5092e-13) | Convergence attained in 2 iterations (rel. change 2.1895e-10) |  |

**TABLE S4.** Regression model summaries with segmented relationship(s) indicating estimated Break-Point (BP) of observed and predicted richness and frequency of well-sampled cells by latitudinal band at global scale and by region. ‘date-specim’ data curation scenario: data obtained after extract only preserved specimens and remove duplicates with identical date of collection.

| **Global Predicted Richness** | **Global Observed Richness** | **Global Frequency of cells** |
| --- | --- | --- |
| BP Est. St.Err | BP Est. St.Err | BP Est. St.Err |
| **psi1.latitudeBands 39 1.463** | **psi1.latitudeBands 39 1.238** | **psi1.latitudeBands 25.499 1.931** |
| Meaningful coefficients of the linear terms: | | |
| Estimate Std. Error t value Pr(>\|t\|) | Estimate Std. Error t value Pr(>\|t\|) | Estimate Std. Error t value Pr(>\|t\|) |
| (Intercept) 92.6426 16.7519 5.530 4.36e-08 *** | (Intercept) 74.1518 16.1474 4.592 5.11e-06 *** | (Intercept) -5.6567 3.4588 -1.635 0.109 |
| latitudeBands 5.5705 0.6342 8.783 < 2e-16 *** | latitudeBands 4.9745 0.6291 7.907 9.00e-15 *** | latitudeBands 1.4261 0.2256 6.322 1.24e-07 *** |
| U1.latitudeBands -14.8726 4.7499 -3.131 NA | U1.latitudeBands -13.7619 3.6560 -3.764 NA | U1.latitudeBands -2.3461 0.3245 -7.230 NA |
| Signif. codes: 0 ‘***’ 0.001 ‘**’ 0.01 ‘*’ 0.05 ‘.’ 0.1 ‘ ’ 1 | | |
| Residual standard error: 123.7 on 780 degrees of freedom | Residual standard error: 113.4 on 780 degrees of freedom | Residual standard error: 7.91 on 43 degrees of freedom |
| **R^2^:** **0.1108, Adj. R^2^: 0.1074** | **R^2^: 0.1049, Adj. R^2^: 0.1014** | **R^2^: 0.5925, Adj. R^2^: 0.5641** |
| Boot restarting based on 10 samples. Last fit: | Boot restarting based on 10 samples. Last fit: | Boot restarting based on 6 samples. Last fit: |
| Convergence attained in 2 iterations (rel. change 7.2973e-10) | Convergence attained in 3 iterations (rel. change 2.0788e-08) | Convergence attained in 3 iterations (rel. change 3.098e-13) |

| **America Predicted Richness** | **America Observed Richness** | **Frequency of cells in American region** |
| --- | --- | --- |
| BP Est. St.Err | BP Est. St.Err | BP Est. St.Err |
| **psi1.latitudeBands 32 1.736** | **psi1.latitudeBands 31.803 1.789** | **psi1.latitudeBands 25.55 1.276** |
| Meaningful coefficients of the linear terms: | | |
| Estimate Std. Error t value Pr(>\|t\|) | Estimate Std. Error t value Pr(>\|t\|) | Estimate Std. Error t value Pr(>\|t\|) |
| (Intercept) 95.9982 18.8791 5.085 4.99e-07 *** | (Intercept) 79.6997 16.9653 4.698 3.29e-06 *** | (Intercept) -4.6754 2.4667 -1.895 0.0649 . |
| latitudeBands 5.0606 0.8032 6.301 5.91e-10 *** | latitudeBands 4.4329 0.7218 6.142 1.53e-09 *** | latitudeBands 1.2630 0.1609 7.851 9.03e-10 *** |
| U1.latitudeBands -11.7771 2.4360 -4.835 NA | U1.latitudeBands -10.4130 2.1891 -4.757 NA | U1.latitudeBands -2.5517 0.2380 -10.723 NA |
| Signif. codes: 0 ‘***’ 0.001 ‘**’ 0.01 ‘*’ 0.05 ‘.’ 0.1 ‘ ’ 1 | | |
| Residual standard error: 110.8 on 574 degrees of freedom | Residual standard error: 99.59 on 574 degrees of freedom | Residual standard error: 5.641 on 42 degrees of freedom |
| **R^2^: 0.07763, Adj. R^2^: 0.07281** | **R^2^: 0.07433, Adj. R^2^:** **0.0695** | **R^2^: 0.7337, Adj. R^2^: 0.7147** |
| Boot restarting based on 6 samples. Last fit: | Boot restarting based on 6 samples. Last fit: | Boot restarting based on 6 samples. Last fit: |
| Convergence attained in 2 iterations (rel. change 1.3953e-08) | Convergence attained in 5 iterations (rel. change 1.6963e-13) | Convergence attained in 4 iterations (rel. change 2.4648e-13) |

| **Europe Predicted Richness** | **Europe Observed Richness** | **Frequency of cells in European region** |
| --- | --- | --- |
| BP Est. St.Err | BP Est. St.Err | BP Est. St.Err |
| **psi1.latitudeBands 29 1.332** | **psi1.latitudeBands 29 1.341** | **psi1.latitudeBands 43 1.429** |
| Meaningful coefficients of the linear terms: | | |
| Estimate Std. Error t value Pr(>\|t\|) | Estimate Std. Error t value Pr(>\|t\|) | Estimate Std. Error t value Pr(>\|t\|) |
| (Intercept) -354.320 127.932 -2.770 0.00618 ** | (Intercept) -380.513 124.488 -3.057 0.00257 ** | (Intercept) -10.7952 3.7336 -2.891 0.00847 ** |
| latitudeBands 27.729 5.400 5.135 7.04e-07 *** | latitudeBands 26.917 5.254 5.123 7.46e-07 *** | latitudeBands 0.6069 0.1232 4.928 6.28e-05 *** |
| U1.latitudeBands -37.366 6.011 -6.216 NA | U1.latitudeBands -36.113 5.849 -6.174 NA | U1.latitudeBands -3.0207 1.3922 -2.170 NA |
| Signif. codes: 0 ‘***’ 0.001 ‘**’ 0.01 ‘*’ 0.05 ‘.’ 0.1 ‘ ’ 1 | | |
| Residual standard error: 120.9 on 187 degrees of freedom | Residual standard error: 117.7 on 187 degrees of freedom | Residual standard error: 5.189 on 22 degrees of freedom |
| **R^2^: 0.1886, Adj. R^2^: 0.1756** | **R^2^: 0.1873, Adj. R^2^: 0.1743** | **R^2^: 0.5689, Adj. R^2^: 0.5101** |
| Boot restarting based on 6 samples. Last fit: | Boot restarting based on 6 samples. Last fit: | Boot restarting based on 6 samples. Last fit: |
| Convergence attained in 1 iterations (rel. change 1.6926e-06) | Convergence attained in 1 iterations (rel. change 3.2668e-06) | Convergence attained in 2 iterations (rel. change 3.5583e-06) |

| **Asia Predicted Richness** | **Asia Observed Richness** | **Frequency of cells in Asian region** |
| --- | --- | --- |
| BP Est. St.Err | BP Est. St.Err | BP Est. St.Err |
| **psi1.latitudeBands 18.456 1.123** | **psi1.latitudeBands 18.388 1.094** | **NA** |
| Meaningful coefficients of the linear terms: | | |
| Estimate Std. Error t value Pr(>\|t\|) | Estimate Std. Error t value Pr(>\|t\|) | Estimate Std. Error t value Pr(>\|t\|) |
| (Intercept) -644.24 266.80 -2.415 0.03433 * | (Intercept) -534.00 217.12 -2.460 0.03171 * | (Intercept) 1.617438 0.475305 3.403 0.0114 * |
| latitudeBands 55.66 16.42 3.390 0.00604 ** | latitudeBands 46.08 13.36 3.449 0.00544 ** | latitudeBands 0.001779 0.015929 0.112 0.9142 |
| U1.latitudeBands -63.22 16.56 -3.816 NA | U1.latitudeBands -52.38 13.48 -3.886 NA |  |
| Signif. codes: 0 ‘***’ 0.001 ‘**’ 0.01 ‘*’ 0.05 ‘.’ 0.1 ‘ ’ 1 | | |
| Residual standard error: 80.44 on 11 degrees of freedom | Residual standard error: 65.46 on 11 degrees of freedom | Residual standard error: 0.534 on 7 degrees of freedom |
| **R^2^: 0.6877, Adj. R^2^: 0.6025** | **R^2^: 0.6951, Adj. R^2^: 0.612** | **R^2^: 0.001779, Adj. R^2^: -0.1408** |
| Boot restarting based on 6 samples. Last fit: | | |
| Convergence attained in 2 iterations (rel. change 2.2222e-13) | Convergence attained in 2 iterations (rel. change 1.1745e-11) |  |

**TABLE S5.** Regression model summaries with segmented relationship(s) indicating estimated Break-Point (BP) of observed and predicted richness and frequency of well-sampled cells by latitudinal band at global scale and by region. ‘date-1970’ data curation scenario: data obtained after remove duplicates with identical date of collection and collected after 1970.

| **Global Predicted Richness** | **Global Observed Richness** | **Global Frequency of cells** |
| --- | --- | --- |
| BP Est. St.Err | BP Est. St.Err | BP Est. St.Err |
| **psi1.latitudeBands 40.65 0.793** | **psi1.latitudeBands 40.216 0.856** | **psi1.latitudeBands 31.057 1.316** |
| Meaningful coefficients of the linear terms: | | |
| Estimate Std. Error t value Pr(>\|t\|) | Estimate Std. Error t value Pr(>\|t\|) | Estimate Std. Error t value Pr(>\|t\|) |
| (Intercept) 52.4514 18.0086 2.913 0.00369 ** | (Intercept) 27.4453 16.8302 1.631 0.103 | (Intercept) -7.3004 2.5338 -2.881 0.00628 ** |
| latitudeBands 6.5963 0.6152 10.722 < 2e-16 *** | latitudeBands 6.3718 0.5749 11.083 <2e-16 *** | latitudeBands 1.2853 0.1301 9.876 2.11e-12 *** |
| U1.latitudeBands -25.5641 5.9237 -4.316 NA | U1.latitudeBands -24.0768 5.5361 -4.349 NA | U1.latitudeBands -2.7157 0.2978 -9.119 NA |
| Signif. codes: 0 ‘***’ 0.001 ‘**’ 0.01 ‘*’ 0.05 ‘.’ 0.1 ‘ ’ 1 | | |
| Residual standard error: 120.5 on 747 degrees of freedom | Residual standard error: 112.6 on 747 degrees of freedom | Residual standard error 5.896 on 41degrees of freedom |
| **R^2^: 0.152, Adj. R^2^: 0.1488** | **R^2^: 0.1562, Adj. R^2^:**  **0.1528** | **R^2^: 0.7618, Adj. R^2^ : 0.7443** |
| Boot restarting based on 6 samples. Last fit: | | |
| Convergence attained in 6 iterations (rel. change 2.8838e-13) | Convergence attained in 2 iterations (rel. change 1.5521e-12) | Convergence attained in 2 iterations (rel. change 3.0305e-15) |

| **America Predicted Richness** | **America Observed Richness** | **Frequency of cells in American region** |
| --- | --- | --- |
| BP Est. St.Err | BP Est. St.Err | BP Est. St.Err |
| **psi1.latitudeBands 28 2.01** | **psi1.latitudeBands 28 1.896** | **psi1.latitudeBands 27.45 1.333** |
| Meaningful coefficients of the linear terms: | | |
| Estimate Std. Error t value Pr(>\|t\|) | Estimate Std. Error t value Pr(>\|t\|) | Estimate Std. Error t value Pr(>\|t\|) |
| (Intercept) 79.688 22.687 3.513 0.000497 *** | (Intercept) 64.799 21.002 3.085 0.00218 ** | (Intercept) -2.9504 1.9371 -1.523 0.136 |
| latitudeBands 4.688 1.025 4.575 6.43e-06 *** | latitudeBands 4.064 0.991 4.101 5.02e-05 *** | latitudeBands 0.8036 0.1131 7.105 1.34e-08 *** |
| U1.latitudeBands -7.722 1.806 -4.276 NA | U1.latitudeBands -6.867 1.531 -4.485 NA | U1.latitudeBands -1.8292 0.1865 -9.806 NA |
| Signif. codes: 0 ‘***’ 0.001 ‘**’ 0.01 ‘*’ 0.05 ‘.’ 0.1 ‘ ’ 1 | | |
| Residual standard error: 95.59 on 384 degrees of freedom | Residual standard error: 83.95 on 384 degrees of freedom | Residual standard error: 4.172 on 40 degrees of freedom |
| **R^2^: 0.06441, Adj. R^2^: 0.0571** | **R^2^: 0.0634, Adj. R^2^: 0.05617** | **R^2^: 0.7124, Adj. R^2^: 0.7124** |
| Boot restarting based on 8 samples. Last fit: | Boot restarting based on 7 samples. Last fit: | Boot restarting based on 6 samples. Last fit: |
| Convergence attained in 2 iterations (rel. change 1.6128e-08) | Convergence attained in 2 iterations (rel. change ) | Convergence attained in 2 iterations (rel. change 7.4673e-12) |

| **Europe Predicted Richness** | **Europe Observed Richness** | **Frequency of cells in Europe region** |
| --- | --- | --- |
| BP Est. St.Err | BP Est. St.Err | BP Est. St.Err |
| **psi1.latitudeBands 40 0.994** | **psi1.latitudeBands 39.527 0.973** | **psi1.latitudeBands 41.621 0.773** |
| Meaningful coefficients of the linear terms: | | |
| Estimate Std. Error t value Pr(>\|t\|) | Estimate Std. Error t value Pr(>\|t\|) | Estimate Std. Error t value Pr(>\|t\|) |
| (Intercept) 85.164 50.999 1.670 0.0959 | (Intercept) 36.207 49.391 0.733 0.464 | (Intercept) -13.83234 2.73105 -5.065 2.83e-05 *** |
| latitudeBands 6.893 1.549 4.450 1.17e-05 *** | latitudeBands 7.375 1.500 4.916 1.38e-06 *** | latitudeBands 0.87070 0.09216 9.447 6.83e-10 *** |
| U1.latitudeBands -23.877 6.414 -3.723 NA | U1.latitudeBands -25.225 6.211 -4.061 NA | U1.latitudeBands -4.04051 0.81046 -4.985 NA |
| Signif. codes: 0 ‘***’ 0.001 ‘**’ 0.01 ‘*’ 0.05 ‘.’ 0.1 ‘ ’ 1 | | |
| Residual standard error: 124.9 on 339 degrees of freedom | Residual standard error: 121 on 339 degrees of freedom | Residual standard error: 3.707 on 26 degrees of freedom |
| **R^2^: 0.07598, Adj. R^2^: 0.0678** | **R^2^: 0.08857, Adj. R^2^: 0.08051** | **R^2^: 0.8042, Adj. R^2^: 0.7816** |
| Boot restarting based on 10 samples. Last fit: | Boot restarting based on 8 samples. Last fit: | Boot restarting based on 6 samples. Last fit: |
| Convergence attained in 2 iterations (rel. change 2.2736e-08) | Convergence attained in 4 iterations (rel. change 1.1803e-12) | Convergence attained in 3 iterations (rel. change 3.8914e-11) |

| **Asia Predicted Richness** | **Asia Observed Richness** | **Frequency of cells in Asian region** |
| --- | --- | --- |
| BP Est. St.Err | BP Est. St.Err | BP Est. St.Err |
| **psi1.latitudeBands 18.361 1.226** | **psi1.latitudeBands 18.289 1.176** | **NA** |
| Meaningful coefficients of the linear terms: | | |
| Estimate Std. Error t value Pr(>\|t\|) | Estimate Std. Error t value Pr(>\|t\|) | Estimate Std. Error t value Pr(>\|t\|) |
| (Intercept) -604.46 282.90 -2.137 0.04842 * | (Intercept) -502.00 227.11 -2.210 0.04199 * | (Intercept) 1.710563 0.590344 2.898 0.0159 * |
| latitudeBands 52.35 17.41 3.006 0.00837 ** | latitudeBands 43.42 13.98 3.106 0.00679 ** | latitudeBands -0.001451 0.018395 -0.079 0.9387 |
| U1.latitudeBands -60.59 17.55 -3.453 NA | U1.latitudeBands -50.27 14.09 -3.569 NA |  |
| Signif. codes: 0 ‘***’ 0.001 ‘**’ 0.01 ‘*’ 0.05 ‘.’ 0.1 ‘ ’ 1 | | |
| Residual standard error: 85.3 on 16 degrees of freedom | Residual standard error: 68.48 on 16degrees of freedom | Residual standard error: 0.6829 on 10 degrees of freedom |
| **R^2^: 0.5933, Adj. R^2^: 0.517** | **R^2^: 0.6094, Adj. R^2^: 0.5361** | **R^2^: 0.0006219, Adj. R^2^: -0.09932** |
| Boot restarting based on 6 samples. Last fit: | | |
| Convergence attained in 2 iterations (rel. change 4.4878e-14) | Convergence attained in 2 iterations (rel. change 3.8949e-12) |  |
